# Iodine fortification of plant-based dairy and fish alternatives: the effect of substitution on iodine intake based on a market survey in the UK

**DOI:** 10.1017/S0007114522001052

**Published:** 2023-03-14

**Authors:** Katie Nicol, Eva-Leanne Thomas, Anne P. Nugent, Jayne V. Woodside, Kathryn H. Hart, Sarah C. Bath

**Affiliations:** 1 Department of Nutritional Sciences, Faculty of Health and Medical Sciences, University of Surrey, Guildford, UK; 2 Institute for Global Food Security, School of Biological Sciences, Queens University Belfast, Northern Ireland, UK; 3 Centre for Public Health, Queens University Belfast, Belfast, UK

**Keywords:** Iodine, Milk, Cheese, Yoghurt, Plant-based, Fish, Calcium

## Abstract

Milk, dairy products, and fish are the main sources of iodine in the UK. Plant-based products are increasingly popular, especially with young women, which may affect iodine intake as they are naturally low in iodine; this is concerning as iodine is required for fetal brain development. We, aimed to (i) assess the iodine fortification of products sold as alternatives to milk, yoghurt, cheese and fish through a cross-sectional survey of UK retail outlets in 2020, and (ii) model the impact of substitution with such products on iodine intake, using portion-based scenarios. We identified 300 products, including plant-based alternatives to: (i) milk (*n* 146); (ii) yoghurt (*n* 76); (iii) cheese (*n* 67) and (iv) fish (*n* 11). After excluding organic products (*n* 48), which cannot be fortified, only 28 % (*n* 29) of milk alternatives and 6 % (*n* 4) of yoghurt alternatives were fortified with iodine, compared with 88 % (*n* 92) and 73 % (*n* 51), respectively, with Ca. No cheese alternative was fortified with iodine, but 55 % were fortified with Ca. None of the fish alternatives were iodine fortified. Substitution of three portions of dairy product (milk/yoghurt/cheese) per day with unfortified alternatives would reduce the iodine provided by 97·9 % (124 *v*. 2·6 µg) and substantially reduce the contribution to the adult intake recommendation (150 µg/d; 83 *v*. 1·8 %). Our study highlights that the majority of plant-based alternatives are not iodine fortified and that the use of unfortified alternatives put consumers at risk of iodine deficiency.

Iodine, as a component of thyroid hormones, is required for thyroid function. Thyroid hormones are crucial for the regulation of metabolism, growth and neurodevelopment. Iodine deficiency can lead to thyroid enlargement, or goitre, inadequate thyroid hormone production and a spectrum of clinical disorders^(1)^. Iodine deficiency during pregnancy can have implications for the developing child – severe deficiency can result in cretinism and intellectual impairments^(2)^ and even mild-to-moderate deficiency has been linked to lower IQ, reading and spelling ability^(3)^. It is becoming increasingly clear that adequate iodine intake is essential even before pregnancy to ensure optimal iodine stores in the thyroid^(4,5)^. Therefore, all women of childbearing age should ensure a sufficient intake of iodine (i.e. 150 µg/d^(2)^); however, this age group is more likely to avoid rich sources of iodine such as fish and dairy^(6,7)^ and more likely to try a plant-based diet^(8)^. Furthermore, although previously believed to be iodine replete, women of childbearing age in the UK are now classified as iodine insufficient according to the latest data from the National Diet and Nutrition Survey (NDNS); iodine status in this group is below the WHO threshold for iodine sufficiency in the general population^(9)^ (median urinary iodine concentration of 97 µg/l and threshold for adequacy is 100 µg/l)^(10)^.

Iodine status in any age group is dependent on individual food choice as, unlike many countries, the UK does not have a salt-iodisation programme^(11)^. In the UK, milk and other dairy products contribute 32 % of the total dietary intake of adults (according to NDNS data), with semi-skimmed milk being the main contributor (13 %)^(9)^. During pregnancy and lactation, studies have shown that milk alone contributes 40 % and 38 % of the dietary intakes of iodine, respectively^(12,13)^, and in pregnancy, other dairy products contribute 31 % of dietary iodine intake^(13)^. Fish is a rich source of iodine, but it contributes less to overall population iodine intake (10 % according to NDNS adult data^(9)^) as fish is a less popular food choice, and consumption is generally lower, especially in young women^(6,9)^. However, on a per-portion basis, fish and fish products could provide a considerable proportion of daily iodine recommendations. For example, a portion of cod provides approximately 230 µg/140 g portion^(14)^ (150 % of recommended iodine intake for adults).

In recent years, there has been an increased interest in plant-based diets and plant-based alternatives to animal products. In 2020, UK sales of plant-based options were 73 % greater than in 2018^(15)^. Plant-based dairy alternatives have seen the most significant growth in the UK market in recent years, with sales of plant-based milk and cheese alternatives increasing by 107 % and 165 %, respectively, in 2020 compared with 2018^(15)^. Almost a quarter (23 %) of UK consumers used plant-based milk alternatives in 2019, according to research from Mintel^(7)^, up from 19 % in 2018. It appears that young women are driving this trend; some 26 % of women report consuming plant-based milk alternatives, and as many as one-third of 16–24-year-olds now opt for these plant-based alternatives^(7)^. This is a concern for iodine intake as the ingredients used to make plant-based alternatives to fish and dairy products are naturally low in iodine and, unless fortified, will not contribute to iodine intake. Previous UK studies of milk alternatives found that only 6 % of milk-alternative products on the market in 2015 were fortified with iodine^(16)^.

In view of the growing consumer interest in plant-based alternative products and the plant-based market’s rapid evolution and expansion, with new products continually being added, we aimed to survey iodine fortification in the plant-based market. This is important as it informs dietary recommendations and may affect iodine status, particularly in groups already identified as iodine deficient in the UK. Therefore, our study aimed to (i) gather data on iodine fortification of products sold in the UK as alternatives to important iodine sources – milk, dairy products and fish, and (ii) to model the impact that consumption of such products would have on iodine intake. This is the first time that the iodine fortification of products that are sold as alternatives to fish has been examined. We therefore provide information on current iodine fortification of all products that replace the primary sources of dietary iodine in the UK.

## Methods

### Market survey: data collection

A cross-sectional survey of plant-based milk-alternative drinks, yoghurt-, cheese-, and fish-alternative products was conducted in December 2020. The following grocery retailers were surveyed: Tesco, Sainsbury, Asda, Morrisons, Aldi, Lidl, Waitrose and Ocado. These collectively constitute 88 % of the UK supermarket market share^(17)^ and were chosen to reflect the readily available choices to the majority of British shoppers.

To ensure that all available products were identified, we used the retailer’s online shopping website. The online search was first done using the drop-down menu on websites to identify ‘plant-based’, ‘vegan’ or ‘free-from’ categories. To capture all dairy products, and in the case of websites without a drop-down menu, the search bar was used for the following terms: ‘vegan milk’, ‘milk alternative’, ‘plant-based milk’ and ‘dairy-free milk’. The search was repeated, substituting ‘milk’ for either ‘cheese’ or ‘yoghurt’. To identify fish-alternative products, the search terms used were ‘vegan fish’, ‘fish alternative’, ‘fishless’, ‘plant-based fish’ and ‘fish-free’. To capture the most accurate data, each online supermarket was surveyed within a period of 1 to 3 d to avoid any changes in product stock. For supermarkets that did not have an online shopping website (Lidl & Aldi), a researcher went in-store to collect data from the food packaging, including nutritional information and ingredient lists.

Plant-based milk and dairy product alternatives from both the refrigerated and long-life sections were included in the survey, where their intended use was presumed to be similar to cow’s milk. Plant-based fish alternative products from both the refrigerated and frozen sections were included. Products excluded from the survey were (i) flavoured plant-based milk alternatives (e.g. chocolate/strawberry); (ii) products that were not sold as replacements to dairy products (i.e. coconut water and canned coconut milk); (iii) products aimed at children under 1 year old and (iv) products that were part of a composite meal (e.g. plant-based pizza with cheese alternative topping). A full list of products surveyed is provided in online Supplementary Tables S1a–d.

### Market survey: data extraction from product label

Information was taken from the online product information, or product packaging, as appropriate. A screenshot was taken from the retailer/manufacturer’s website, or a photograph of the commercial packaging was taken in store. If a product was sold by more than one retailer, the data were extracted only once. A second researcher checked the data extracted and removed any duplicate products. The product name and brand were recorded. Data on nutrient content were extracted from the nutritional label (per 100 ml or 100 g); this included energy, macronutrients and where available micronutrients including Ca, iodine and vitamins B_2_, B_12_ and D. The ingredient list was also checked for fortification with iodine (and other nutrients). To be classified as an iodine-fortified product, the nutritional information had to list iodine (>0 μg/100 g) and must contain iodine (e.g. potassium iodide/iodate) in the ingredients list.

Milk and yoghurt alternatives were categorised by the main plant ingredient or matrix (e.g. soya/almond). For cheese alternatives, the type of cheese replacement was also noted (i.e. soft/hard cheese). Whether products were fresh or frozen was noted for the fish alternatives. The products were categorised based on whether they were organic, sweetened (for milk/yoghurt) or fortified with at least one micronutrient. We report results for organic and non-organic products separately because organic product regulations do not allow the use of fortificants that are not required by law, meaning that many organic products will not contain micronutrients that non-organic equivalents may have added. Data entries were collated into a Microsoft Excel spreadsheet (Redmond, WA, USA), and values were cross-validated by a second member of the research team.

### Calculation of iodine content/portion

In order to compare the iodine (and other minerals) concentration of the plant-based products to their equivalent animal-based versions, and for the purposes of our dietary modelling, nutrition composition data were obtained from UK food tables for cow’s milk, cheese, yoghurt and fish products, and median values were calculated^(14)^ (see online Supplementary Table S2 for products used for calculating average values). In terms of comparison of the fish products, we selected data from the UK food tables for white fish products that were similar to the plant-based fish alternative products identified in the market survey (i.e. fried or baked coated white fish products, such as breaded fillets or fish fingers). For the purposes of dietary modelling, we calculated the iodine content of white fish and oily fish products separately (see online Supplementary Table S2).

The iodine concentration of the fortified milk and yoghurt products was calculated by averaging the values from the fortified products identified in the market survey (online Supplementary Table S1).

In cases where micronutrient data were unavailable from the nutritional label, such as in non-fortified products, values were derived for each product based on previous analysis of unfortified plant-based milk alternatives^(16)^. For products not included in the previous analysis, values were derived factorially for each product, based on composition data from UK food tables^(14)^. This calculation was done using the percentage of the characterising ingredient (e.g. soya or pea) as UK Quantitative Ingredients Declaration Guidelines state that the characterising ingredient must be declared as a percentage of the final food product^(18)^. This percentage was then used to compute nutritional values per 100 g. For example, a product containing 3 % almonds without additional fortification was considered nutritionally equivalent to 3 g of unprocessed almonds per 100 ml of milk-alternative or 100 g of cheese-, yoghurt- or fish alternative (i.e. 2 µg iodine/100 g of almonds). Details of ingredient weighting used for calculations are provided in online Supplementary Table S3.

We calculated iodine per average portion on the basis that one adult portion of milk, yoghurt, cheese and fish (or their plant-based equivalent) was 200 g, 150 g, 30 g and 140 g, respectively^(19)^.

### Modelling iodine intake with dietary scenarios

We used information on availability of iodine-fortified alternative products from the market survey to model the effect of substituting both dairy and fish with alternative products. Iodine intake from three portions of plant-based dairy alternatives (milk, yoghurt and cheese alternatives) was modelled against a reference dairy scenario of three portions of conventional dairy products. Although there are no official UK recommendations for the number of portions of milk and dairy products to consume each day, we chose three portions as a daily target to reflect guidance from dietitians and other organisations that adults should aim for these amounts to achieve the recommended Ca intake^(20,21)^.

We modelled three scenarios based on varying numbers of fortified plant-based alternatives: Scenario I included two fortified alternatives (milk and yoghurt alternatives) and an unfortified cheese alternative, Scenario II included one fortified alternative (milk alternative) and two unfortified alternatives (yoghurt and cheese alternatives) and Scenario III included three unfortified alternatives (milk, yoghurt and cheese alternatives). We did not model an iodine-fortified cheese product as this was not available on the market.

To model the effect of substituting fish with alternative products, we modelled intake against a reference fish scenario, which was based on the current UK recommendation of two portions of fish/week, one of which is oily^(21)^. Scenario IV was based on two portions of unfortified fish-alternative products (as the market survey did not identify any iodine-fortified versions). The iodine concentration of fish alternative products was estimated from UK food tables using the characterising ingredient (online Supplementary Table S3), and iodine provision was calculated as a daily intake (µg/d).

Iodine intake from the different scenarios was expressed as a percentage of iodine intake recommendations for vulnerable groups in the UK, i.e. young women and pregnant/lactating women, as women of childbearing age are more likely to follow a plant-based diet^(8)^ and try plant-based alternatives^(7)^. We used the EFSA adequate intake (AI) values for adults (150 µg/d) and pregnant/lactating women (200 µg/d)^(1)^. In addition, as children may also follow plant-based diets (especially in families where adults are consuming plant-based alternative products), we also modelled the effect on their iodine intake. As children eat smaller portions than adults, we recalculated iodine per portion using portion sizes of 100 ml of milk, 125 g yoghurt, 20 g cheese and 70 g portions of fish (online Supplementary Table S4); in the absence of official guidance, these portions sizes were based on data from a variety of sources for children^(22–24)^. Iodine intake as a proportion of children’s requirements was based on EFSA iodine intake recommendations for children 1–10 years (90 µg/d), 11–14 years (120 µg/d) and 15–18 years (130 µg/d).

### Data and statistical analysis

All data were checked for normality using the Shapiro–Wilk test and visual inspection of histogram plots, and as data were not normally distributed, we report median and 25th, 75th percentile. A Mann–Whitney *U* test was used to compare the micronutrient concentration of conventional cow’s milk products or fish products with that of the plant-based alternatives. Statistical analyses were performed using SPSS Statistics version 27.0 (IBMCorp.). Significance was set at *P* < 0·05. Plots were generated using R version 4.0.3^(25)^ using packages ggplot2^(26)^, ggpubr^(27)^, ggThemeAssist^(28)^ and ggforce^(29)^.

## Results

Following the exclusion of product duplicates, a total of 146 milk-alternative drinks were identified, which was a 62 % increase from our survey in 2015 (Table 1), with the greatest growth seen in oat-based milk (+300 %) and pea-based milk emerging as a new category. Of the drinks identified in the current survey (Table 2), soya-based drinks were most common (*n* 36, 25 %), followed by almond (*n* 34, 23 %) oat (*n* 32, 22 %) and coconut (*n* 21, 14 %). Overall, 23 % (*n* 34) were sweetened, 28 % (*n* 41) were organic and 72 % (*n* 105) were fortified with at least one micronutrient.

**Table 1.** Changes in product numbers and types of plant-based milk alternatives on the UK market between the 2015 and 2020 surveys

	2015 product survey (*n*)*	2020 product survey (*n*)	% Change
Total products	90	146	62 %
Almond	17	34	100 %
Coconut	7	21	200 %
Other nut	4	12	200 %
Oat	8	32	300 %
Pea	0	6	–
Rice	8	5	−38 %
Soya	46	36	−22 %

* 2015 values taken from previous market survey^(16)^.

The selection criteria for both the 2015 and 2020 surveys included unflavoured milk alternatives available from major retailers.

**Table 2.** Median concentration of iodine in plant-based milk (per 100 ml), yoghurt, cheese and fish alternative products (per 100 g) that are fortified with iodine (Numbers and percentages; median values and range)

	Total products (n)	Total non-organic products (n)	Number fortified with iodine, (n)	Percentage of total fortified with iodine (%)	Percentage of non-organic products fortified with iodine (%)	Iodine concentration of fortified products (µg/100 ml or g)		*P* value*
						Median	Range	
Milk								
All milk alternatives	146	105	29	20	28	24·8	13·0–45·0	0·17
Almond	34	24	5	15	21	24·8	24·7–45·0	
Coconut	21	15	4	19	27	24·7	13·0–30·0	
Oat	32	21	7	22	33	22·5	22·5–30·0	
Other nut	12	8	1	8	13	25·0	NA	
Pea	6	6	4	67	67	26·8	22·5–31·0	
Rice	5	2	0	0	0	NA†	NA†	
Soya	36	29	8	22	28	24·9	22·4–45·0	
Yoghurt								
All yoghurt alternatives	76	70	4	5	6	22·5	22·5–22·5	0·01
Almond	10	10	0	0	0	NA†	NA†	
Coconut	23	19	0	0	0	NA†	NA†	
Cashew	1	0	0	0		NA†	NA†	
Oat	9	9	4	5	6	22·5	22·5–22·5	
Soya	33	32	0	0	0	NA†	NA†	
Cheese								
All cheese alternatives	67	66	0	0	0	NA†	NA†	NA
Almond-based	3	2	0	0	0	NA†	NA†	
Coconut-based	64	64	0	0	0	NA†	NA†	
Fish								
All fish alternatives	11	11	0	0	0	NA^†^	NA^†^	NA

NA: Not applicable.

* *P* value for comparison of iodine content of plant-based alternative with iodine content of cow’s milk product of the same category (iodine values from Table 4); *P* value from Mann–Whitney *U* test.

† Fortified values not available.

A total of seventy-six plant-based yoghurt alternatives were identified (Table 2), with soya being the most common (*n* 33, 43 %) followed by coconut (*n* 23, 30 %). Overall, 77 % (*n* 58) were flavoured and 8 % (*n* 6) were organic. A total of sixty-seven plant-based cheese alternatives were identified, with coconut oil being the base ingredient in 95 % of products (*n* 64; Table 2). Of all the cheese alternatives, 33 % (*n* 22) were replacements for fresh-style cheeses (e.g. cream cheese, mozzarella), 36 % (*n* 24) for cheddar cheese, 12 % (*n* 8) replaced semi-hard cheese and 19 % (*n* 13) were unspecified or other cheese styles (e.g. parmesan). Finally, the survey identified eleven plant-based fish alternatives (alternatives to breaded/coated white fish), of which six products (55 %) were breaded fish fillet or goujon substitutes, four (36 %) were fish finger substitutes and one product (9 %) was a replacement for conventional fishcakes.

### Micronutrient fortification

**Fig. 1.**
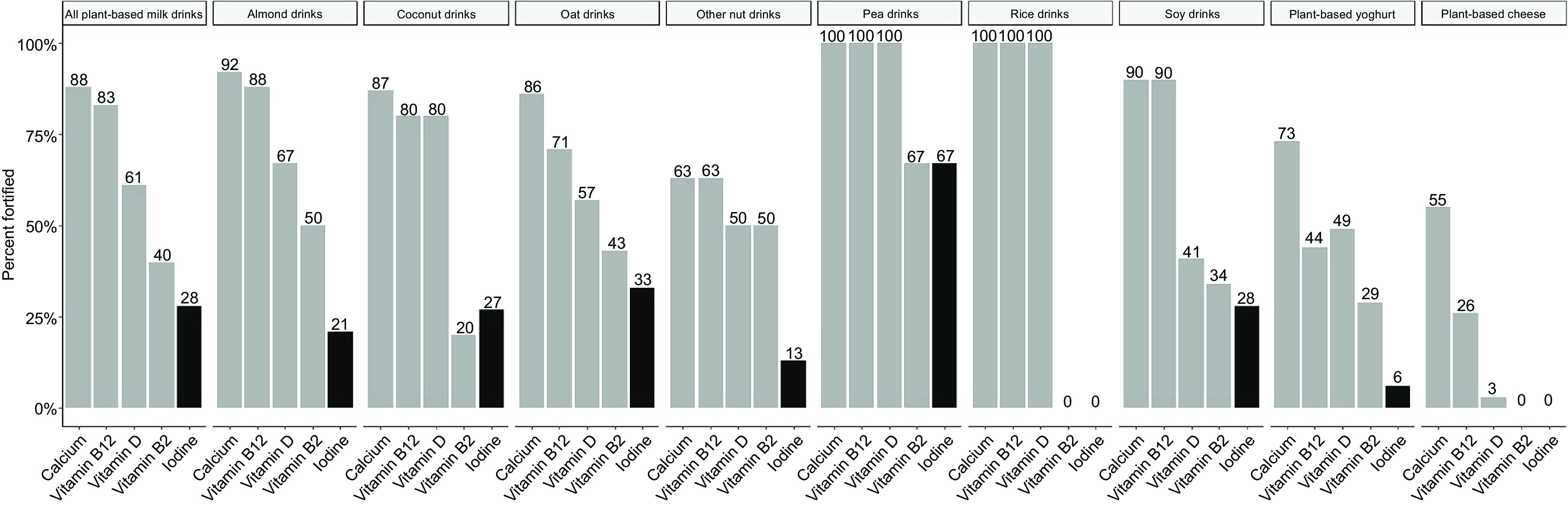
The proportion of non-organic plant-based milk and dairy alternative products within each category that fortified with micronutrients according to the product packaging.

Of all the 146 milk alternatives, only 20 % (*n* 29) were fortified with iodine (Table 2). Fig. 1 shows the percentage of non-organic drinks (*n* 105) fortified with micronutrients; the percentage fortified with iodine (28 %) was considerably lower than the percentage fortified Ca (88 %, *n* 92), vitamin B_12_ (83 %, *n* 87), vitamin D (61 % *n* 65) and vitamin B_2_ (40 %, *n* 43). When examining iodine fortification by milk-alternative type, pea-based drinks were the most frequently fortified (67 %), while none of the rice-based milk-alternative drinks were fortified with iodine (Fig. 1 and Table 2).

**Fig. 2.**
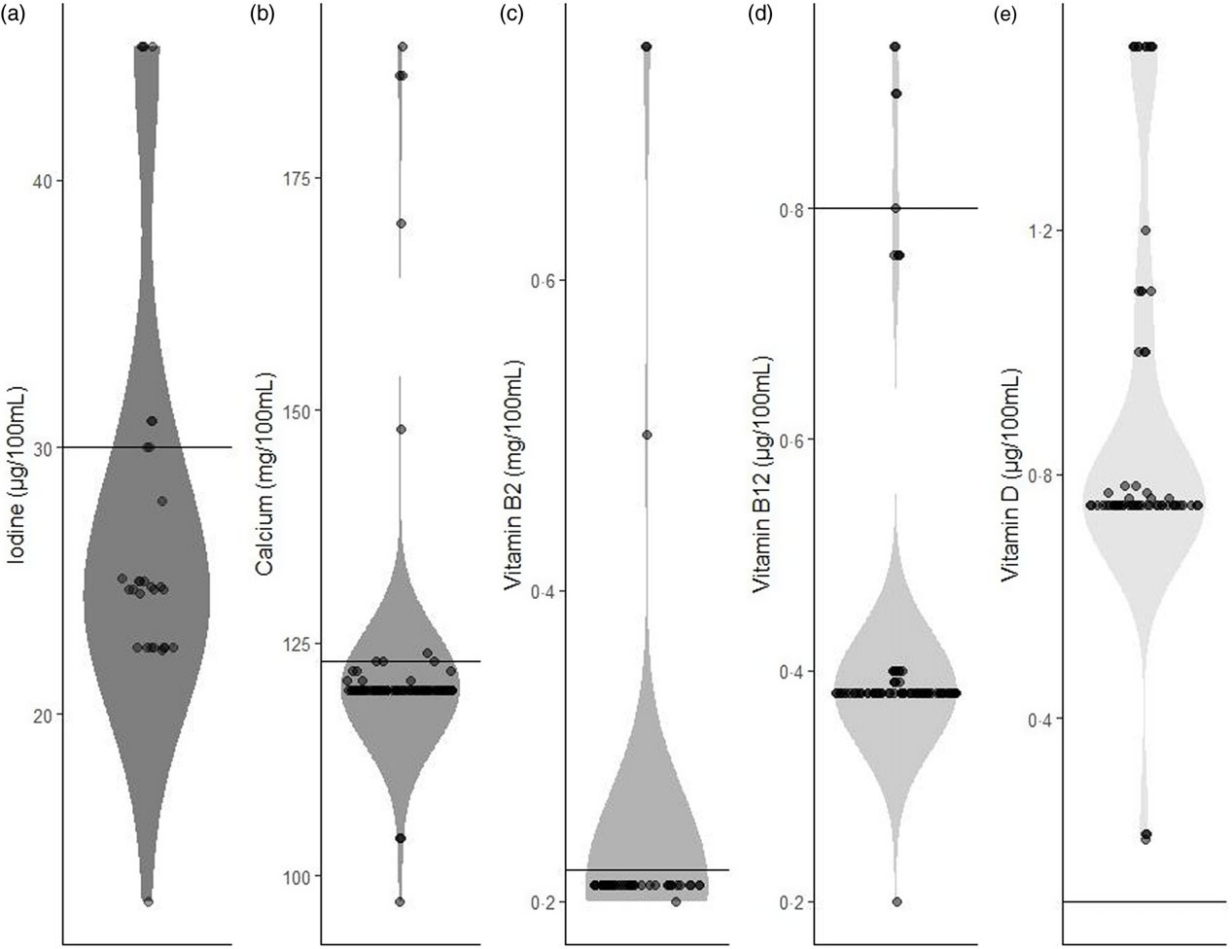
Violin plot to show the range of fortification of five micronutrients in fortified plant-based milk alternative products available on the UK market. (a) Iodine; (b) calcium; (c) Vitamin B_2_; (d) Vitamin B_12_; (e) Vitamin D. Horizontal line indicates median cow’s milk value for each micronutrient (iodine: 30 µg/100 ml calcium: 123 mg/100 ml, Vitamin B_2_:0·22 mg/100 ml, Vitamin B_12_:0·8 µg/100 ml, Vitamin D: 0·1 µg/100 ml).

The iodine fortification of the milk alternatives ranged from 13 to 45 µg/100 ml (Table 2; Fig. 2), and the median value (24·8 µg/100 ml; Table 2) was lower than that of cow’s milk (median 30·0 µg/100 ml, range 20–41 µg/100 ml), although the difference was not significant (*P* = 0·17; Table 2). A 200 ml portion of a fortified milk-alternative would provide approximately 50 µg (Table 3) or 33·3 % of adult iodine recommendations.

**Table 3.** Median iodine content per adult portion of cow’s milk products, fish and plant-based alternative products (fortified and unfortified)

Category	Product	Adult portion size^(19)^ (g)	Iodine content (µg/100 g)	Iodine/portion (µg/portion)
Milk	Cow’s milk	200	30·0*	60·0
	Fortified milk-alternative		24·8†	49·6
	Unfortified milk alternative		0·56‡	1·12
Yoghurt	Yoghurt from cow’s milk	150	36·5*	55·0
	Fortified yoghurt alternative		22·5†	33·75
	Unfortified yoghurt alternative		0·84‡	1·26
Cheese	Cheese from cow’s milk	30	29·5*	9·0
	Fortified cheese alternative		NA	NA
	Unfortified cheese alternative		0·8‡	0·25
Fish	White fish	140	106·0*	148·4
	Oily fish		14·0*	19·6
	Fortified fish alternative		NA	NA
	Unfortified fish alternative		0·5‡	0·7

NA: fortified product not available on the market.

* Median values from UK food tables for milk (skimmed, semi-skimmed, 1 % and whole cow’s milk), yoghurt (low fat, Greek, Fromage Frais, whole), cheese (soft, hard, cheese spread, processed) and fish (white and oily) (see detail in online Supplementary Table S2).

† Median value from fortified product identified in our market survey (no fortified cheese product available on the market; online Supplementary Table S1).

‡ Calculated value based on iodine content of ingredient list (online Supplementary Table S3).

Ca fortification of plant-based milk alternatives ranged from 97 to 189 mg/100 ml, and the median fortification was not significantly different to that of cow’s milk (Table 4). The median vitamin B_2_ and B_12_ concentrations of the fortified milk alternatives were equivalent to 95 % and 48 %, respectively, of that found in conventional cow’s milk (the vitamin B_12_ value being significantly lower than cow’s milk). However, the vitamin D content was significantly higher in the plant-based milk alternatives than cow’s milk (median 0·75 *v*. 0·1 µg/100 ml, respectively).

**Table 4.** Median and range of concentrations of calcium, vitamins B_2_, B_12_ and D in fortified plant-based milk (per 100 ml), yoghurt, cheese and fish alternative products (per 100 g) that are fortified with each nutrient. Nutrient values for cow’s milk products and fish products are given for comparison (Numbers and percentages; median values and range)

			Calcium				Vitamin B_2_				Vitamin B_12_				Vitamin D			
				Concentration (mg/100 ml or g)				Concentration (mg/100 ml or g)				Concentration (µg/100 ml or g)				Concentration (µg/100 ml or g)		
	Total products (n)	Total non-organic products (n)	Number fortified (n)	Median	Range	*P* value*	Number fortified (n)	Median	Range	*P* value*	Number fortified (n)	Median	Range	*P* value*	Number fortified (n)	Median	Range	*P* value*
Milk																		
Cow’s milk	NA	NA	NA	123†	102–133†	0·10	NA	0·22†	0·17–0·24†	<0·001	NA	0·80†	0·20–0·90†	<0·001	NA	0·10†	0·0–0·10†	<0·001
Milk alternatives	146	105	92	120	97–189		43	0·21	0·20–0·75		87	0·38	0·20–0·94		65	0·75	0·20–1·50	
Yoghurt																		
Cow’s milk yoghurt	NA	NA	NA	140†	87–200†	0·01	NA	0·21†	0·13–0·37†	0·90	NA	0·30†	0·00–0·40†	<0·001	NA	0·10†	0·0–0·10†	<0·001
Yoghurt alternatives	76	70	51	120	55–240		14	0·21	0·21–0·24		31	0·38	0·30–0·42		34	0·75	0·6–2·00	
Cheese																		
Cow’s milk cheese	NA	NA	NA	544†	76–1025†	<0·001	NA	0·39†	0·19–0·65†	NA	NA	1·20†	0·30–4·10†	<0·001	NA	0·20†	0·0–0·50†	<0·001
Cheese alternatives	67	66	36	185	150–736		0	NA‡	NA‡		17	2·24	0·38–2·70		2	0·98	0·75–1·20	
Fish																		
Fish (white) products	NA	NA	NA	51†	23–180†	NA	NA	0·08†	0·06–0·13†	NA	NA	1·40†	0·30–2·10†	NA	NA	0·25†	0·0–0·50†	NA
Fish alternatives	11	11	0	NA‡	NA‡		0	NA‡	NA‡		0	NA‡	NA‡		0	NA‡	NA‡	

NA: not applicable.

* Comparison of nutrient content of plant-based alternative with that of cow’s milk or fish product of the same category; *P* value from Mann–Whitney *U* test.

† Values from UK food tables (see online Supplementary Table S2).; comparison only with white fish products as oily-fish alternative products were not identified in the market survey.

‡ Fortified values not available.

A low proportion of the non-organic plant-based alternative yoghurt products were fortified with iodine (*n* 4, 6 %), all of which were oat-based yoghurts from the same brand (Table 2). In comparison, 73 % (*n* 51) were fortified with Ca, 49 % fortified with vitamin D and 44 % were fortified with vitamin B_12_. The median iodine concentration of the iodine-fortified yoghurt alternatives (22·5 µg/100 g) was significantly lower (*P* = 0·01) than that of cow’s milk yoghurt (median 36·5 µg/100 g, range 17·0–63·0 µg/100 g). A portion of fortified yoghurt alternative would provide around 34 µg (Table 3) or 22·5 % of the adult daily iodine recommendation. By contrast, the unfortified yoghurt alternative would provide just 1·3 µg of iodine (Table 3).

The Ca-fortified yoghurt alternatives had a significantly lower (*P* = 0·01) Ca content than that of cow’s milk yoghurts (median 120 *v*. 140 mg/100 g), while the vitamin D and vitamin B_12_ content in the fortified yoghurt alternatives was significantly higher than that of cow’s milk (Table 4).

No plant-based cheese alternatives were fortified with iodine, but cheese made from cow’s milk is a source of iodine with a median concentration of 29·5 µg/100 g (range 4·0–72·0 µg/100 g). Unfortified plant-based cheese was estimated to have an iodine concentration of just 0·8 µg/100 g (Table 3). By contrast, 55 % (*n* 36) of non-organic plant-based cheese was fortified with Ca, 26 % (*n* 17) with vitamin B_12_ and 3 % (*n* 2) with vitamin D, none were fortified with vitamin B_2_.

None of the fish-alternative products (*n* 11) were fortified with iodine. The products were all marketed as breaded white fish alternatives, and these fish products have an iodine concentration of 106 µg/100 g (average of fried and baked coated white fish products, e.g. fish fingers, online Supplementary Table S2).

### Dietary scenarios

The changes in iodine intake when substituting dairy or fish with fortified and unfortified plant-based alternatives are shown in Table 5 for adults. The two reference scenarios indicate that combining three portions of dairy/d and two portions of fish/week would provide 99 % (148 µg/d) of the adult recommendation for iodine (150 µg/d); one portion of milk, cheese and yoghurt would provide 83 % (124 µg/d), and two portions of fish would provide 16 % (24 µg/d) of the daily adult recommendation.

**Table 5.** Dietary scenarios used to model the implications for daily iodine intake when replacing cow’s milk dairy products and fish with plant-based alternatives products

		Iodine provided* (µg/d)	Iodine provided as percentage of iodine intake recommendation	
Scenario			Adult (% of 150 µg/d)^(1)^	Pregnancy/lactation (% of 200 µg/d)^(1)^
Reference dairy scenario	Three portions of dairy per day from cow’s milk: one portion each of milk, yoghurt and cheese	124·0	83·0 %	62·0 %
I	Two portions of fortified plant-based dairy: one portion of fortified milk alternative, one portion of fortified yoghurt alternative and one portion of unfortified cheese alternative	83·6	55·7 %	41·8 %
II	One portion of fortified plant-based dairy: one portion of fortified milk alternative, one portion of unfortified yoghurt and one portion of unfortified cheese alternative	51·1	34·1 %	25·6 %
III	Three portions of unfortified plant-based dairy alternatives: one portion each of unfortified milk, yoghurt and cheese alternative products	2·6	1·8 %	1·3 %
Reference fish scenario	Two portions of fish/week: one portion of white fish and one portion of oily fish	24·0†	16·0 %	12·0 %
IV	Two portions of fish alternative products/week	0·2†	0·13 %	0·10 %

* Calculated using data from Table 3.

† Adjusted to daily value by dividing by 7.

In Scenario I, consuming one portion each of a fortified plant-based milk alternative and yoghurt alternative and one portion of an unfortified plant-based cheese alternative, would provide 55·7 % (83·6 µg/d) of the iodine required for adequate intake for adults and 41·8 % during pregnancy. Replacing three portions of dairy with unfortified plant-based alternatives (Scenario III) would result in a 97·9 % reduction in daily iodine intake (2·6 v. 124 µg/d) and a substantially lower contribution to the  iodine intake recommendations for adults (1·8 % *v*. 83 %) and pregnant women (1·3 % *v*. 62 %; Table 5). Compared with the reference scenario for fish (24 µg/d), Scenario IV would contribute only 0·2 µg/d and a low contribution to the adult and pregnancy iodine intake recommendations (0·13 % and 0·1 % respectively; Table 5).

Similar results were found when modelling the impact on children’s iodine intake; the reference dairy scenario alone provides between 62·7 % and 90·6 % of the iodine intake recommendations (for children 15–18 years and 1–10 years, respectively; online Supplementary Table S5). Replacing three portions of dairy with unfortified versions (Scenario III) would result in a 97·9 % reduction in iodine provided, and contribution to intake recommendations would be just 1·3–1·9 % for children (online Supplementary Table S5). Scenario II (where just the milk-alternative is fortified) provides 28·9 % of iodine recommendations for children 1–10 years and 20 % for children 15–18 years (online Supplementary Table S5). The reference fish scenario would provide up to 13·3 % of children’s iodine recommendations, compared with just 0·1 % with unfortified plant-based alternatives (online Supplementary Table S5).

## Discussion

Our study has shown that plant-based milk- and dairy-alternatives were poor sources of iodine compared to cow’s milk. At the time of this survey, most milk-alternatives on the UK market (80 %) were not fortified with iodine. Even after focusing only on non-organic (i.e. those that could potentially be fortified), only 28 % of non-organic milk-alternative drinks were fortified with iodine, compared with 88 % with Ca. Furthermore, only one brand of yoghurt-alternative products was iodine fortified, and none of the cheese or fish alternative products were fortified with iodine at the time of our survey.

Our survey updates two recent UK market surveys (one in 2019 and one in early 2020) that included evaluation of the iodine fortification of milk-alternative drinks. The survey in 2019 included a small number of outlets and only identified eighty-two drinks (*v*. 146 in our survey), of which just six (7·3 %) were fortified with iodine. The survey by Clegg *et al.* in July 2020 also identified just 6 (of 136 drinks) fortified with iodine but also included cheese and yoghurt alternatives in the survey; interestingly at that time none of the yoghurt or cheese products were fortified with iodine, while 6 months later we found a slight improvement in the yoghurt-alternative market, as 6 % were fortified with iodine in our survey. As the first study in the UK to also include products sold as alternatives to fish, we are able to provide a comprehensive summary of all the products marketed as a replacement for the main dietary sources of iodine in the UK.

Cow’s milk and fish, mainly white fish, are the primary sources of iodine in the UK. Our study suggests that adults consuming three portions of dairy each day and two portions of fish/week, as per general advice^(20,21)^, would consume approximately 148 µg of iodine/d, close to the threshold for adequate intake and the mean iodine intake of British adults (159 µg/d from NDNS data^(9)^). However, if consumers were to replace these products with plant-based alternatives, the likelihood of selecting a product fortified with iodine is low. Consequently, the most likely dietary scenario is that those who substitute dairy and fish products with plant-based alternatives will consume unfortified varieties (our Scenarios III and IV) due to the high percentage of milk and yoghurt alternative products not fortified with iodine. Switching from dairy and fish products to unfortified plant-based alternatives would result in the iodine provided by these food groups dropping by 97 %. One portion of an iodine-fortified milk alternative and unfortified plant-based cheese and yoghurt options (Scenario II) would contribute to one-third of adults’, and 20–29 % of children’s’, daily iodine intake recommendation. Therefore, our results indicate that the fortification of plant-based milk alternatives alone is not enough to ensure adequacy, all plant-based dairy and fish alternatives should be fortified with iodine to ensure those following a plant-based diet and consuming plant-based alternatives can still achieve adequate iodine intake.

Our results are not just relevant to the UK, but would be applicable in any country where milk, dairy products and fish are the main dietary sources of iodine. This is the case in many European countries, for example Norway, Finland and Spain and also in the USA, Australia and New Zealand. Our findings concur with previous market surveys conducted in countries other than the UK. There have been regional market surveys published in recent years on plant-based milk alternatives from Australia^(30)^, Canada^(31)^, India^(32)^, Italy^(33)^, Switzerland^(34)^, the USA^(35)^ and Norway^(36)^. While most of these studies do not include assessments of iodine, the surveys conducted in Australia and Norway found very few products were fortified with iodine^(30)^ and the study in Italy found that none of the products surveyed were fortified with micronutrients^(33)^. In addition to these market surveys, studies in Norway^(36)^ and the USA^(37)^ have specifically analysed the iodine content of milk-alternative drinks available in those countries. Both studies found that the iodine content was low, unless fortified.

Although plant-based alternatives to iodine-rich foods have been available for many years, there has been a rapid rise in the availability of plant-based options, indeed we noted an increase in products since our 2015 study^(16)^. As a result, understanding the implications of replacing these products with plant-based alternatives is of great importance, particularly in terms of iodine intake. The low frequency of iodine fortification of these plant-based dairy- and fish alternatives is becoming an issue as more people adopt vegan or plant-based dietary patterns^(16)^. Vegans have been found to have marginal or iodine-deficient intakes^(38)^, and therefore it is important that they select iodine-fortified products where possible. This is important as younger women are the most likely to try a plant based or vegan diet^(8)^. Recent figures from Years 9 to 11 of the UK NDNS indicate that women of childbearing age are currently classified as iodine insufficient^(9)^. A switch from conventional products to unfortified plant-based alternatives may exacerbate this problem. Indeed, analysis of data from plant-based milk consumers in the UK found that those consuming exclusively plant-based milk alternatives were more likely to have an insufficient iodine intake, and low iodine status, than those exclusively consuming cow’s milk^(39)^. The situation could be further worsened if other dietary sources of iodine are replaced by unfortified alternatives, as was modelled in the current study.

Compared with the plant-based milk alternatives available in 2015^(16)^, our results show an increase in the prevalence of iodine fortification (20 % *v*. 6 %). Nonetheless, the overall frequency of iodine fortification remains low compared with the number of plant-based milk alternatives fortified with other micronutrients such as Ca (88 %) and B_12_ (83 %). Additionally, the type of beverage selected by consumers is expected to influence how likely they are to choose an iodine-fortified product. For example, none of the rice beverages surveyed were fortified with iodine, while pea beverages were the most likely to be fortified. However, only six pea beverages were included in the survey and therefore make up a small proportion of the market, so, in terms of contribution to population, iodine intake from pea-based beverages is still likely to be low.

Another important consideration is whether the products are organic, as there are notable differences in the micronutrient content of organic *v*. non-organic plant-based alternatives, as organic products cannot be fortified with micronutrients. We found that 27 % of milk alternatives, 8 % of yoghurt alternatives and 2 % of cheese alternatives were organic plant-based products and therefore had no added iodine. Many plant-derived ingredients used in milk and dairy alternatives (e.g. oat/rice) are naturally low in micronutrients such as iodine^(16,37)^, and as a result, fortification of the product is a significant determinant of micronutrient composition. Subsequently, organic and other non-fortified plant-based milks and dairy alternatives are a poor nutritional substitute for cow’s milk and dairy products. In organic dairy farming, cows consume more pasture and lower amounts of concentrate feed and in the past, organic milk was found to have a lower iodine concentration compared with traditional cow’s milk in the UK, particularly in the summer months^(40)^. However, more recent analysis of UK milk has found no difference in iodine concentration between organic and conventional farms and shows that organic milk is a rich source of iodine^(41)^.

The median amount of iodine added to fortified milk-alternative drinks (24·8 µg/100 ml) was at the lower end of the range of iodine concentration of UK cow’s milk (20–41 µg/100 ml). There was variation in the amount of iodine added to plant-based milk alternatives, with some products fortified a lower iodine concentration than cow’s milk, while other brands contained a concentration towards to the top of the cow’s milk range. It is important to note that there is considerable variation in milk-iodine values according to season, and also that UK cow’s milk has a high iodine concentration relative to other countries^(42)^. Indeed, the median iodine fortification of plant-based drinks in our study would be higher than milk available in some countries, such as the Netherlands and New Zealand^(42)^. Most fortified versions of milk alternatives in our study had a concentration that would provide 30 % of the adult RNI in a 200 ml portion, and when we included a fortified yoghurt in our modelling scenario, 56 % of the adult requirement was provided (Scenario II).

Consumers should be encouraged to select iodine-fortified dairy alternatives where possible, to help meet iodine requirements. However, many consumers of plant-based alternatives may not be able to select an iodine-fortified version (i.e. depending on availability in different retail outlets), particularly if they prefer to use the organic non-fortified options. Even if a fortified version were used, whether consumers would meet their dietary requirements with these fortified milk-alternative products would depend on the quantity consumed and whether other sources of iodine (e.g. fish, eggs and other dairy products) were present in the diet. For individuals who are following a strict plant-based or vegan diet, many of these food sources are not appropriate. There are few plant-based sources of iodine, as although seaweed is rich in iodine, it is an unreliable source as the iodine content is highly variable^(43)^ and consumption of some seaweed species (e.g. kelp) could result in excessive iodine intake^(43,44)^. Therefore, a suitable iodine-containing supplement (not providing more than the daily recommended iodine intake and not a kelp supplement) may be required for consumers with limited dietary sources of iodine.

The lack of iodine fortification in plant-based alternatives is a concern as several studies have emphasised the lack of knowledge and awareness surrounding the importance of iodine and iodine-rich foods^(13,45)^, which may add to the problem of inadequate dietary intake in the UK. Women of childbearing age have been shown to have a limited understanding of dietary sources of iodine and the consequences of iodine deficiency^(45)^. Over half of mothers surveyed in Scotland were unable to identify correct sources of iodine, commonly mistaking salt (21 %) and vegetables (54 %) as iodine-rich foods^(13)^, and this lack of knowledge has been associated with low iodine intakes^(45)^. Considering the lack of knowledge surrounding iodine-rich foods, it is likely that consumers may not realise the importance of selecting a plant-based alternative to dairy or fish that is fortified with iodine, especially if iodine is traditionally not declared on the nutritional labelling of these products. Emphasising this point, a recent UK study found that only 8 % of cow’s milk surveyed declared iodine on the nutritional labelling compared with 59 % that declared Ca content^(46)^. If consumers are not aware that iodine is found in milk, they will not know that they need to ensure adequate iodine from other sources. It is crucial to raise awareness among consumers of the importance of the micronutrients found in dairy and fish, which may also help consumers make more informed, healthier choices. Product formulations may change with time, and fortification levels were observed to vary between brands and even within different types of the same brand (i.e. sweetened *v*. unsweetened); this is confusing for the consumer.

This study has some limitations both in terms of the market survey and the dietary modelling. First, we have not analysed the iodine concentration of the products on the market and instead have had to rely on the nutritional information supplied by the manufacturer or have imputed values based on the nutritional content of the main ingredient. Second, we did not include products sold in health food stores, independent stores or cafes in this survey. Although we attempted to capture a complete list of products available on the UK market, products sold in health food stores, independent stores or cafes and flavoured plant-based milk alternatives were excluded from this survey; it would be worthwhile monitoring the market for new products that might be fortified, and hence including these categories in future study. Finally, and importantly, our survey is cross-sectional, and therefore the results are limited to the current market. As manufacturers and consumers become more aware of iodine, more brands may begin to fortify their dairy and fish alternatives. Our modelling using dietary scenarios has limitations in that it is based on an optimal, healthy diet, as we used dietary intake recommendations for fish and dairy intake; it is likely that this does not reflect actual intake for all population groups.

### Further research

Further research is required to understand the role of plant-based milk and other dairy alternative products on iodine intake and status in the UK. Future work could examine the impact of substituting plant-based alternatives on actual intakes, using population-level data and considering the current market share of iodine-fortified products. Furthermore, a future study to investigate the bioavailability of iodine added to plant-based alternatives is needed to know whether the iodine-fortified versions provide iodine in a way that is equivalent to cows’ milk. Consideration of the specification of iodine in the product, for example, if iodine is present in seaweed, is also important.

### Conclusions

The current study has highlighted major differences in the iodine content between plant-based alternatives and conventional dairy and fish products. A small proportion of products was fortified with iodine, and so cannot be considered nutritionally equivalent to milk, dairy products and fish in terms of iodine intake. Individuals who consume unfortified alternatives in place of conventional dairy and fish may be at risk of iodine deficiency. The rising popularity of a plant-based diet and lifestyle will fuel the consumer demand for plant-based alternatives to foods from animal origins^(47)^. Manufacturers of such alternative products should fortify their products with an appropriate amount of iodine.

## Supplementary material

For supplementary material accompanying this paper visit https://doi.org/10.1017/S0007114522001052. click here to view supplementary material
