# Supplementary material for: Iodine fortification of plant-based dairy and fish alternatives: the effect of substitution on iodine intake based on a market survey in the UK
Source: Br J Nutr. 2022 Apr 4;129(5):832–42. doi: 10.1017/S0007114522001052 (PMC9975780; doi:10.1017/S0007114522001052)
Supplement: Supplementary file 1 [file S0007114522001052sup001.docx]

**Supplementary tables for Iodine fortification of plant-based dairy and fish alternatives – the effect of substitution on iodine intake based on a market survey in the UK**

K. Nicol^1^, E. Thomas^1^, A. Nugent^2^, J. Woodside^3^, K. Hart^1^, S.C. Bath^1^

[Supplementary Table S1a: plant-based milk alternatives on the UK market with micronutrient fortification (per 100 mL). 2](#_Toc92728117)

[Supplementary Table S1b: plant-based yogurt alternatives on UK market and fortification level (per 100g) 8](#_Toc92728118)

[Supplementary Table S1c: plant-based cheese alternatives on the UK market with micronutrient fortification (per 100g). 12](#_Toc92728119)

[Supplementary Table S1d: plant-based fish alternatives on the UK market with micronutrient fortification (per 100g). 16](#_Toc92728120)

[Supplementary Table S2: Nutritional composition (per 100g) of animal-based milk, yoghurt, cheese and fish from UK Food Tables [1]. 17](#_Toc92728121)

[Supplementary Table S3: ingredients used for modelling from UK food tables [1] 22](#_Toc92728122)

[References 22](#_Toc92728123)

Supplementary Table S1a: plant-based milk alternatives on the UK market with micronutrient fortification (per 100 mL). Survey completed in December 2020. Flavoured drinks and those only available in health-food shops/cafes not included.

| **Matrix** | **Brand** | **Product description** | **Processing** | **Organic** | **Sweetened** | **Iodine (µg/100mL)** | **Calcium (mg/100mL)** | **Vitamin B2 (mg/100mL)** | **Vitamin B12 (µg/100mL)** | **Vitamin D (µg/100 mL)** |
| --- | --- | --- | --- | --- | --- | --- | --- | --- | --- | --- |
| Almond (n=34) | Aldi | Original | Long life | N | Y | - | 120 | - | 0.38 | - |
|  | Aldi | Unsweetened | Long life | N | N | - | 120 | - | 0.38 | - |
|  | Alpro | Original | Chilled | N | Y | - | 120 | 0.21 | 0.38 | 0.75 |
|  | Alpro | Original | Long life | N | Y | - | 120 | 0.21 | 0.38 | 0.75 |
|  | Alpro | Unsweetened | Long life | N | N | - | 120 | 0.21 | 0.38 | 0.75 |
|  | Alpro | Roasted unsweetened | Chilled | N | N | - | 120 | 0.21 | 0.38 | 0.75 |
|  | Alpro | Roasted Almond | Long life | N | N | - | 120 | 0.21 | 0.38 | 0.75 |
|  | Asda | Unsweetened | Chilled | N | N | 24.7 | 121 | 0.21 | 0.38 | 0.75 |
|  | Asda | Unsweetened | Long life | N | N | 24.7 | 121 | 0.21 | 0.38 | 0.75 |
|  | Asda | Sweetened | Long life | N | Y | 24.8 | 121 | 0.21 | 0.38 | 0.75 |
|  | Blue Diamond | Barista Blend | Long life | N | N | - | - | - | - | - |
|  | Blue Diamond | Original | Long life | N | Y | - | 120 | - | 0.38 | 0.75 |
|  | Blue Diamond | Unsweetened | Long life | N | N | - | 120 | - | 0.38 | 0.75 |
|  | Califa Farms | Unsweetened | Chilled | N | N | - | 189 | - | - | - |
|  | Drink Bruce | Original | Chilled | Y | N | - | - | - | - | - |
|  | Innocent | Original | Chilled | N | N | - | - | - | - | - |
|  | Just Free (Lidl) | Sweetened | Long life | Y | Y | - | - | - | - | - |
|  | Just Free (Lidl) | Unsweetened | Long life | Y | N | - | - | - | - | - |
|  | Marks & Spencer | Sweetened | Long life | N | Y | 45 | 120 | - | 0.76 | 1.5 |
|  | Marks & Spencer | Unsweetened | Long life | N | N | 45 | 120 | - | 0.76 | 1.5 |
|  | Morrisons | Sweetened | Long life | N | Y | - | 120 | - | 0.4 | - |
|  | Morrisons | Unsweetened | Long life | N | N | - | 120 | - | 0.4 | - |
|  | Plenish | Unsweetened | Chilled | Y | N | - | - | - | - | - |
| Almond cont. | Plenish | 6% Almond | Chilled | Y | N | - | - | - | - | - |
|  | Provamel | Original | Long life | Y | N | - | - | - | - | - |
|  | Rude Health | Original | Chilled | Y | N | - | - | - | - | - |
|  | Rude Health | Barista Blend | Chilled | Y | N | - | - | - | - | - |
|  | Rude Health | Original | Long life | Y | N | - | - | - | - | - |
|  | Rude Health | Ultimate | Long life | Y | N | - | - | - | - | - |
|  | Sainsbury's | Unsweetened | Long life | N | N | - | 120 | 0.21 | 0.38 | 0.75 |
|  | Tesco | Sweetened | Chilled | N | Y | - | 120 | 0.21 | 0.38 | 0.75 |
|  | Tesco | Unsweetened | Chilled | N | N | - | 120 | 0.21 | 0.38 | 0.75 |
|  | Tesco | Sweetened | Long life | N | Y | - | 120 | 0.21 | 0.38 | 0.75 |
|  | Tesco | Unsweetened | Long life | N | N | - | 120 | 0.21 | 0.38 | 0.75 |
| Coconut (n=21) | Alpro | Coconut & Almond | Chilled | N | Y | - | 120 | 0.21 | 0.38 | 0.75 |
|  | Alpro | Original | Chilled | N | N | - | 120 | - | 0.38 | 0.75 |
|  | Alpro | Unsweetened | Chilled | N | N | - | 120 | - | 0.38 | 0.75 |
|  | Alpro | Original | Long life | N | N | - | 120 | - | 0.38 | 0.75 |
|  | Asda | Original | Chilled | N | N | 24.7 | 122 | - | 0.38 | - |
|  | Asda | Original | Long life | N | N | 24.7 | 122 | - | 0.38 | 0.76 |
|  | Drink Bruce | Coconut & Almond | Chilled | Y | N | - | - | - | - | - |
|  | Innocent | Original | Chilled | N | N | - | - | - | - | - |
|  | Koko | Original | Long life | N | N | - | 120 | - | 0.38 | 0.75 |
|  | Koko | Unsweetened | Long life | N | N | - | 120 | - | 0.38 | 0.75 |
|  | Koko | Super | Chilled | N | N | 13 | 170 | 0.5 | 0.2 | 1.2 |
|  | Lucy Bee | Original | Long life | Y | N | - | - | - | - | - |
|  | Marks & Spencer | Original | Long life | N | N | 30 | 120 | - | 0.9 | 0.75 |
|  | Plenish | Original | Chilled | Y | N | - | - | - | - | - |
|  | Rebel Kitchen | Semi-skimmed | Chilled | Y | N | - | - | - | - | - |
| Coconut cont. | Rebel Kitchen | Whole | Chilled | Y | N | - | - | - | - | - |
|  | Rude Health | Original | Chilled | Y | N | - | - | - | - | - |
|  | Tesco | Sweetened | Chilled | N | Y | - | 120 | - | 0.38 | 0.75 |
|  | UFC | Unsweetened | Long life | N | Y | - | 97.3 | - | - | 0.2 |
|  | Valsoia | Coconut & Almond | Long life | N | Y | - | 120 | 0.21 | 0.38 | 1.5 |
|  | Vita Coco | Unsweetened | Chilled | N | N | - | - | - | - | - |
| Oat (n=32) | Aldi | Original | Long life | N | Y | - | 120 | - | 0.38 | - |
|  | Aldi | Unsweetened | Long life | N | N | - | - | - | - | - |
|  | Alpro | Unsweetened | Long life | N | N | - | 120 | 0.21 | 0.38 | 0.75 |
|  | Asda | Original | Chilled | N | N | 25.1 | 124 | - | 0.39 | - |
|  | Asda | Original | Long life | N | N | 28 | 148 | - | 0.8 | - |
|  | Califa Farms | Barista Blend | Chilled | N | N | - | 104 | - | - | - |
|  | Califa Farms | Original | Chilled | N | N | - | 104 | - | - | 0.75 |
|  | Drink Bruce | Unsweetened | Chilled | Y | N | - | - | - | - | - |
|  | Good Hemp | Oat & hemp | Long life | N | N | - | - | - | - | - |
|  | Innocent | Original | Chilled | N | N | - | - | - | - | - |
|  | Jord | Oat & hemp | Chilled | Y | N | - | - | - | - | - |
|  | Just Free (Lidl) | Unsweetened | Long life | Y | N | - | - | - | - | - |
|  | Marks & Spencer | Original | Long life | N | N | 30 | 120 | - | 0.9 | 0.75 |
|  | Minor Figures | Barista Blend | Long life | N | N | - | 120 | - | - | - |
|  | Minor Figures | Barista Blend | Long life | Y | N | - | - | - | - | - |
|  | MOMA | Barista Blend | Long life | N | N | - | 120 | 0.21 | 0.38 | 1 |
|  | MOMA | Unsweetened | Long life | N | N | - | 120 | 0.21 | 0.38 | 1 |
|  | Morrisons | Original | Long life | N | N | - | 120 | - | 0.4 | - |
|  | Oatly | Barista Blend | Long life | N | N | 22.5 | 120 | 0.21 | 0.38 | 1.1 |
|  | Oatly | Semi-skimmed | Chilled | N | N | 22.5 | 120 | 0.21 | 0.38 | 1.1 |
|  | Oatly | Whole | Chilled | N | N | 22.5 | 120 | 0.21 | 0.38 | 1.1 |
| Oat cont. | Oatly | Original | Chilled | Y | N | - | - | - | - | - |
|  | Oatly | Original | Long life | Y | N | - | - | - | - | - |
|  | Oatly | Skinny | Chilled | N | N | 22.5 | 120 | 0.21 | 0.38 | 1.1 |
|  | Plenish | Unsweetened | Chilled | Y | N | - | - | - | - | - |
|  | Plenish | Original | Chilled | Y | N | - | - | - | - | - |
|  | Provamel | Original | Long life | Y | N | - | - | - | - | - |
|  | Provitamil | Original | Chilled | N | N | - | 120 | - | 0.38 | 0.75 |
|  | Rude Health | Barista Blend | Long life | Y | N | - | - | - | - | - |
|  | Rude Health | Unsweetened | Chilled | Y | N | - | - | - | - | - |
|  | Sainsbury's | Original | Long life | N | N | - | 120 | 0.21 | 0.38 | 0.75 |
|  | Valsoia | Original | Long life | N | N | - | 120 | 0.21 | 0.38 | 1.5 |
| Other-Nut (n=12) | Alpro | Hazelnut | Chilled | N | N | - | 120 | 0.21 | 0.38 | 0.75 |
|  | Alpro | Hazelnut | Long life | N | N | - | 120 | 0.21 | 0.38 | 0.75 |
|  | Alpro | Cashew | Long life | N | Y | - | 120 | - | 0.38 | - |
|  | Asda | Hazelnut | Chilled | N | Y | 25 | 123 | 0.21 | 0.38 | 0.77 |
|  | Good Hemp | Seed | Long life | N | N | - | - | - | - | - |
|  | Good Hemp | Seed | Long life | N | N | - | - | - | - | - |
|  | Innocent | Hazelnut | Chilled | N | N | - | - | - | - | - |
|  | Plenish | Cashew | Chilled | Y | N | - | - | - | - | - |
|  | Plenish | Cashew | Chilled | Y | N | - | - | - | - | - |
|  | Plenish | Hazelnut | Chilled | Y | N | - | - | - | - | - |
|  | Rude Health | Tiger Nut | Long life | Y | N | - | - | - | - | - |
|  | Valsoia | Walnut | Long life | N | Y | - | 120 | 0.21 | 0.38 | 1.5 |
| Pea (n=6) | Mighty Pea | Original | Chilled | N | Y | 31 | 186 | - | 0.94 | 0.78 |
|  | Mighty Pea | Unsweetened | Chilled | N | N | 31 | 186 | - | 0.94 | 0.78 |
| Pea cont. | Qwrkee | Sweetened | Long life | N | Y | 22.5 | 120 | 0.75 | 0.38 | 0.21 |
|  | Qwrkee | Unsweetened | Long life | N | N | 22.5 | 120 | 0.75 | 0.38 | 0.21 |
|  | Sproud | Barista Blend | Long life | N | Y | - | 120 | 0.21 | 0.38 | 1 |
|  | Sproud | Unsweetened | Long life | N | N | - | 120 | 0.21 | 0.38 | 1.5 |
| Rice (n=5) | Alpro | Original | Long life | N | N | - | 120 | - | 0.38 | 0.75 |
|  | Rice Dream | Original | Long life | Y | N | - | - | - | - | - |
|  | Rice Dream | Original with calcium | Long life | N | N | - | 120 | - | 0.38 | 0.75 |
|  | Riso Scotti | Original | Long life | Y | N | - | - | - | - | - |
|  | Rude Health | Brown Rice | Long life | Y | N | - | - | - | - | - |
| Soya (n=36) | Aldi | Original | Long life | N | Y | - | 120 | - | 0.38 | - |
|  | Aldi | Unsweetened | Long life | N | N | - | 120 | - | 0.38 | - |
|  | Alpro | Barista Blend | Long life | N | N | - | 120 | - | 0.38 | - |
|  | Alpro | Junior Growing Up | Long life | N | Y | 24.5 | 120 | 0.21 | 0.38 | 1.5 |
|  | Alpro | My Cuppa | Chilled | N | Y | - | - | - | - | - |
|  | Alpro | Unsweetened | Long life | Y | N | - | - | - | - | - |
|  | Alpro | Original | Chilled | N | Y | 22.5 | 120 | 0.21 | 0.38 | 0.75 |
|  | Alpro | Original | Long life | N | Y | 22.4 | 120 | - | 0.38 | - |
|  | Alpro | Plant Protein | Long life | N | N | - | 120 | - | 0.38 | - |
|  | Alpro | Light | Chilled | N | N | - | 120 | 0.21 | 0.38 | 0.75 |
|  | Alpro | Unsweetened | Chilled | N | N | - | 120 | 0.21 | 0.38 | 0.75 |
|  | Asda | Sweetened | Chilled | N | Y | 25 | 123 | 0.2 | 0.39 | 0.77 |
|  | Asda | Sweetened | Long life | N | Y | 25 | 123 | - | 0.39 | - |
|  | Asda | Unsweetened | Long life | N | N | - | - | - | - | - |
|  | Asda | Unsweetened | Long life | N | N | 24.8 | 122 | - | 0.39 | - |
|  | Bonsoy | Sweetened | Long life | Y | Y | - | - | - | - | - |
| Soya cont. | Growers Harvest | Unsweetened | Long life | N | N | - | - | - | - | - |
|  | Just Free (Lidl) | Original | Long life | N | Y | - | 120 | - | 0.38 | - |
|  | Marks & Spencer | Sweetened | Long life | N | Y | 45 | 120 | - | 0.76 | 1.5 |
|  | Marks & Spencer | Unsweetened | Long life | N | N | 45 | 120 | - | 0.76 | 0.76 |
|  | Morrisons | Unsweetened | Long life | Y | N | - | - | - | - | - |
|  | Morrisons | Original | Long life | N | Y | - | 120 | - | 0.4 | - |
|  | Morrisons | Unsweetened | Long life | N | N | - | 120 | - | 0.4 | - |
|  | Morrisons | Sweetened | Long life | N | Y | - | 120 | - | 0.4 | - |
|  | Morrisons | Unsweetened | Long life | N | N | - | 120 | - | 0.4 | - |
|  | Morrisons | Sweetened | Long life | N | Y | - | 120 | - | 0.4 | - |
|  | Morrisons | Unsweetened | Long life | N | N | - | 120 | - | 0.4 | - |
|  | Provamel | Original | Long life | Y | N | - | - | - | - | - |
|  | Rude Health | Original | Long life | Y | N | - | - | - | - | - |
|  | Tesco | Sweetened | Long life | Y | Y | - | - | - | - | - |
|  | Tesco | Unsweetened | Long life | Y | N | - | - | - | - | - |
|  | Tesco | Unsweetened | Chilled | N | N | - | 120 | 0.21 | 0.38 | 0.75 |
|  | Valsoia | Light | Long life | N | Y | - | 120 | 0.21 | 0.38 | 1.5 |
|  | Valsoia | Original with calcium | Long life | N | Y | - | 120 | 0.21 | 0.38 | 1.5 |
|  | Waitrose | Sweetened | Long life | N | Y | - | 120 | 0.21 | 0.38 | 0.75 |
|  | Waitrose | Unsweetened | Long life | N | N | - | 120 | 0.21 | 0.38 | 0.75 |

Dashed line indicates product not fortified as information not provided on the nutritional label.

Supplementary Table S1b: plant-based yogurt alternatives on UK market and fortification level (per 100g). Survey completed in December 2020; products only available in health-food shops/cafes not included.

| **Matrix** | **Brand** | **Product description** | **Flavoured** | **Organic** | **Sweetened** | **Iodine  (µg/100 g)** | **Calcium (mg/100 g)** | **Vitamin B2 (mg/100 g)** | **Vitamin B12 (µg/100 g)** | **Vitamin D (µg/100 g)** |
| --- | --- | --- | --- | --- | --- | --- | --- | --- | --- | --- |
| Almond   (n=10) | Beleaf | Mango Passionfruit | Y | N | N | - | - | - | - | - |
|  | Beleaf | Berries | Y | N | N | - | - | - | - | - |
|  | Danone | Plain | N | N | N | - | - | - | - | - |
|  | Nush | Plain | N | N | N | - | - | - | - | - |
|  | Nush | Strawberry | Y | N | N | - | - | - | - | - |
|  | Nush | Raspberry | Y | N | N | - | - | - | - | - |
|  | Nush | Kids Strawberry Tubes | Y | N | N | - | - | - | - | - |
|  | Nush | Kids Blueberry Tubes | Y | N | N | - | - | - | - | - |
|  | Nush | Peach Melba | Y | N | N | - | - | - | - | - |
|  | Nush | Caramel & Hibiscus | Y | N | N | - | - | - | - | - |
| Coconut  (n=23) | Andros | Peach | Y | N | Y | - | 120 | - | - | - |
|  | Andros | Strawberry | Y | N | Y | - | 120 | - | - | - |
|  | Coconut Collabratovie | Blueberry | Y | N | Y | - | - | - | - | - |
|  | Coconut Collabratovie | Raspberry | Y | N | Y | - | - | - | - | - |
|  | Coconut Collabratovie | Alphonso Mango Passion Fruit | Y | N | Y | - | 55 | - | - | 0.8 |
|  | Coconut Collabratovie | Natural | N | N | N | - | - | - | - | - |
|  | Coconut Collabratovie | Mango & Passion Fruit | Y | N | Y | - | - | - | - | - |
|  | Coconut Collabratovie | Madagascan Vanilla | Y | N | N | - | - | - | - | - |
|  | Coconut Collabratovie | Blueberry Yoghurt | Y | N | Y | - | - | - | - | - |
| Coconut cont. | Little Coco Nutters | Strawberry & Banana | Y | N | N | - | 130 | - | 0.4 | 2 |
|  | Little Coco Nutters | Mango & Passion Fruit | Y | N | Y | - | 130 | - | 0.4 | 2 |
|  | Co Yo | Natural | N | Y | N | - | 0 | - | 0 | 0 |
|  | Co Yo | Strawberry | Y | Y | N | - | 0 | - | 0 | 0 |
|  | Koko | Plain | N | N | N | - | 160 | - | 0.38 | 0.75 |
|  | Koko | Vanilla | Y | N | Y | - | 128 | - | 0.38 | 0.75 |
|  | Koko | Strawberry | Y | Y | Y | - | 128 | - | 0.38 | 0.75 |
|  | Koko | Raspberry | Y | N | Y | - | 128 | - | 0.38 | 0.75 |
|  | Koko | Peach & Passionfruit | Y | Y | Y | - | 128 | - | 0.38 | 0.75 |
|  | Marks & Spencer | Mango & Passion Fruit | Y | N | N | - | - | - | - | - |
|  | Marks & Spencer | Blueberry | Y | N | N | - | - | - | - | - |
|  | Marks & Spencer | Plain |  | N | N | - | - | - | - | - |
|  | Marks & Spencer | Vanilla | Y | N | N | - | - | - | - | - |
|  | Oykos | Chocolate Stracciatella | Y | N | Y | - | - | - | - | - |
| Cashew (n=1) | Nush | Natural | N | Y | N | - | - | - | - | - |
| Oat  (n=9) | Activia | Mango & Flaxseeds | Y | N | Y | - | 120 | - | - | - |
|  | Alpro | Plain | N | N | N | - | 0 | - | - | - |
|  | Light & Free | Mango & Passion Fruit | Y | N | N | - | 149 | - | - | - |
|  | Light & Free | Vanilla | Y | N | N | - | 147 | - | - | - |
|  | Light & Free | Raspberry & White Peach | Y | N | N | - | 151 | - | - | - |
|  | Oatly | Greek Style | N | N | N | 22.5 | 131 | - | 0.38 | 1.1 |
|  | Oatly | Plain | N | N | N | 22.5 | 143 | - | 0.38 | 1.1 |
| Oat cont. | Oatly | Strawberry | Y | N | Y | 22.5 | 122 | - | 0.38 | 1.1 |
|  | Oatly | Blueberry | Y | N | Y | 22.5 | 125 | - | 0.38 | 1.1 |
| Soya (n=33) | Alpro | Blueberry | Y | N | Y | - | 120 | 0.21 | 0.38 | 0.75 |
|  | Alpro | Cherry | Y | N | Y | - | 120 | 0.21 | 0.38 | 0.75 |
|  | Alpro | Natural | N | N | Y | - | 120 | - | 0.38 | 0.75 |
|  | Alpro | Zero Sugars | N | N | N | - | 120 | - | 0.38 | 0.75 |
|  | Alpro | Greek Style | N | N | Y | - | 120 | - | 0.38 | 0.75 |
|  | Alpro | Blackberry Raspberry & Cranberry | N | N | Y | - | 120 | 0.21 | 0.38 | 0.75 |
|  | Alpro | Greek Style Strawberry & Raspberry | Y | N | Y | - | 96 | - | 0.38 | - |
|  | Alpro | No Bits Strawberry Peach | Y | N | Y | - | 120 | 0.21 | 0.38 | 0.75 |
|  | Alpro | Strawberry | Y | N | Y | - | 120 | 0.21 | 0.38 | 0.75 |
|  | Alpro | Strawberry | Y | N | Y | - | 120 | 0.21 | 0.38 | 0.75 |
|  | Alpro | Vanilla | Y | N | Y | - | 120 | 0.21 | 0.38 | 0.75 |
|  | Alpro | Coconut | N | N | Y | - | 120 | - | 0.38 | 0.75 |
|  | Alpro | Peach & Exotic Fruit | Y | N | Y | - | 120 | 0.21 | 0.38 | 0.75 |
|  | Alpro | Greek Style Blueberry | Y | N | Y | - | 108 | - | 0.3 | 0.6 |
|  | Alpro | Greek Style Passion Fruit | Y | N | Y | - | 96 | - | 0.3 | 0.6 |
|  | Alpro | Greek Style Mango | Y | N | Y | - | 96 | - | - | 0.6 |
|  | Alpro | No Added Sugar Mango | Y | N | N | - | 120 | 0.21 | 0.38 | 0.75 |
|  | Alpro | Greek Style Vanilla | Y | N | Y | - | 120 | 0.21 | 0.38 | 0.75 |
|  | Alpro | Greek Style Granola Vanilla | Y | N | Y | - | 107 | - | - | - |
|  | Alpro | More Fruit No Added Sugars Cherry | Y | N | N | - | 120 | 0.21 | 0.38 | 0.75 |
|  | Alpro | Kids Strawberry & Banana | Y | N | Y | - | 133 | 0.24 | 0.42 | 0.83 |
|  | Alpro | Kids Peach and Pear | Y | N | Y | - | 133 | 0.24 | 0.42 | 0.83 |
| Soya cont. | Alpro | Kids Mango | Y | N | Y | - | 120 | - | - | - |
|  | Asda | Lemon | Y | N | N | - | 120 | - | - | - |
|  | Asda | Strawberry | Y | N | Y | - | 120 | - | - | - |
|  | Asda | Plain | N | N | N | - | 120 | - | - | 0.75 |
|  | Provamel | Plain | N | Y | N | - | - | - | - | - |
|  | Tesco | Strawberry Fromage Frais | Y | N | Y | - | 240 | - | - | 1.5 |
|  | Tesco | Apricot Fromage Frais | Y | N | Y | - | 240 | - | - | - |
|  | Tesco | Greek Style | Y | N | N | - | 120 | - | - | - |
|  | Tesco | Natural | N | N | N | - | 120 | - | - | - |
|  | Tesco | Passion Fruit Raspberry | Y | N | Y | - | 120 | - | - | - |
|  | Tesco | Mango | Y | N | Y | - | 120 | - | - | - |

Dashed line indicates product not fortified as information not provided on the nutritional label.

Supplementary Table S1c: plant-based cheese alternatives on the UK market with micronutrient fortification (per 100g). Survey completed in December 2020 omitting those only available through health-food shops.

| **Matrix** | **Brand** | **Product description** | **Type** | **Organic** | **Iodine  (µg/ 100g)** | **Calcium (mg/100g)** | **Vitamin B2 (mg/100g)** | **Vitamin B12 (µg/100g)** | **Vitamin D (µg/100g)** |
| --- | --- | --- | --- | --- | --- | --- | --- | --- | --- |
| Almond   (n=3) | Bonsan | Crème Original | Soft /cream cheese | Y | - | - | - | - | - |
|  | Nush | Chive Soft Cheese Alternative | Soft /cream cheese | N | - | - | - | - | - |
|  | Nush | Soft Cheese Alternative | Soft /cream cheese | N | - | - | - | - | - |
| Coconut  (n=64) | Asda | Garlic & Chive Cheddar Alternative | Cheddar | N | - | 150 | - | - | - |
|  | Asda | Garlic & Herb Soft Cheese Alternative | Soft /cream cheese | N | - | 150 | - | - | - |
|  | Asda | Grated Cheddar Alternative | Cheddar | N | - | 150 | - | - | - |
|  | Asda | Grated Mozzarella Alternative | Mozzarella | N | - | 150 | - | - | - |
|  | Asda | Mature Cheddar Alternative | Cheddar | N | - | 150 | - | - | - |
|  | Asda | Sliced Mature Cheddar Alternative | Cheddar | N | - | 150 | - | - | - |
|  | Asda | Soft Cheese Alternative | Soft /cream cheese | N | - | 150 | - | - | - |
|  | Applewood | Slices | Not Specified | N | - | 282 | - | 2.7 | - |
|  | Applewood | Smoky Cheese Alternative | Not Specified | N | - | 282 | - | 2.7 | - |
|  | Follow Your Heart | Gouda Alternative Slices | Gouda | N | - | - | - | - | - |
|  | Follow Your Heart | Cheddar Style Slices | Cheddar | N | - | - | - | - | - |
|  | Green Vie | Mozzarella Flavour Block | Mozzarella | N | - | - | - | 2.5 | - |
| Coconut cont. | Koko | Cream Cheese Alternative | Soft /cream cheese | N | - | 200 | - | 0.38 | 0.75 |
|  | Koko | Cheddar Alternative | Cheddar | N | - | 736 | - | 0.8 | 1.2 |
|  | Morrisons | Cheddar Alternative | Cheddar | N | - | - | - | - | - |
|  | Morrisons | Garlic & Chive Cheddar Alternative | Cheddar | N | - | - | - | - | - |
|  | Morrisons | Garlic & Herb Soft Cheese | Soft /cream cheese | N | - | - | - | - | - |
|  | Morrisons | Mature Cheddar Alternative | Cheddar | N | - | - | - | - | - |
|  | Morrisons | Soft Cheese Alternative | Soft /cream cheese | N | - | - | - | - | - |
|  | Morrisons | Smoked Cheddar Alternative | Cheddar | N | - | - | - | - | - |
|  | Morrisons | Cheddar Alternative Slices | Cheddar | N | - | - | - | - | - |
|  | Morrisons | Mozzarella Alternative Slices | Mozzarella | N | - | - | - | - | - |
|  | Morrisons | Red Leicester Alternative | Red Leicester | N | - | - | - | - | - |
|  | Morrisons | Red Leicester Alternative Slices | Red Leicester | N | - | - | - | - | - |
|  | Mexicana | Mexican Style Cheese | Not Specified | N | - | 244 | - | 2.5 | - |
|  | Nurish | Camembert Alternative | Camembert | N | - | - | - | - | - |
|  | Ocado | Cheddar Alternative | Cheddar | N | - | - | - | - | - |
|  | Ocado | Grated Cheddar Alternative | Cheddar | N | - | - | - | - | - |
|  | Ocado | Grated Mozzarella Alternative | Mozzarella | N | - | - | - | - | - |
|  | Sainsbury's | Garlic & Herb Soft Cheese Alternative | Soft /cream cheese | N | - | 150 | - | - | - |
|  | Sainsbury's | Cheddar & Onion Alternative | Cheddar | N | - | 150 | - | - | - |
|  | Sainsbury's | Grated Cheddar Alternative | Cheddar | N | - | 150 | - | - | - |
|  | Sainsbury's | Cheddar Alternative | Cheddar | N | - | 150 | - | - | - |
|  | Sainsbury's | Cheddar Alternative Slices | Cheddar | N | - | 150 | - | - | - |
| Coconut cont. | Sainsbury's | Soft Cheese Alternative | Soft /cream cheese | N | - | 150 | - | - | - |
|  | Sainsbury's | Blue-Style Cheese Alternative | Blue-style Cheese | N | - | 150 | - | - | - |
|  | Sainsbury's | Wensleydale-Style Cheese Alternative | Wensleydale-Style Cheese | N | - | 150 | - | - | - |
|  | Sheese | Mozzarella Alternative | Mozzarella | N | - | 150 | - | - | - |
|  | Sheese | Cheddar Spread Alternative | Cheddar | N | - | 150 | - | - | - |
|  | Sheese | Garlic & Herb Soft Cheese Alternative | Soft /cream cheese | N | - | 150 | - | - | - |
|  | Sheese | Soft Cheese Alternative | Soft /cream cheese | N | - | 150 | - | - | - |
|  | Sheese | Cheddar Alternative With Jalapeno & Chilli | Cheddar | N | - | 150 | - | - | - |
|  | Sheese | Smoked German Style | Not Specified | N | - | 150 | - | - | - |
|  | Tesco | Grated Mozzarella Alternative | Mozzarella | N | - | 150 | - | - | - |
|  | Tesco | Cheddar Alternative With Jalapeno & Chilli | Cheddar | N | - | 150 | - | - | - |
|  | Tesco | Alternative With Chilli | Not Specified | N | - | 150 | - | - | - |
|  | Tesco | Halloumi Alternative | Halloumi | N | - | 150 | - | - | - |
|  | Tesco | Hard Cheese Alternative | Not Specified | N | - | 150 | - | - | - |
|  | Tesco | Soft Cheese Alternative | Soft /cream cheese | N | - | 150 | - | - | - |
|  | Tesco | Garlic & Herb Soft Cheese Alternative | Soft /cream cheese | N | - | 150 | - | - | - |
|  | Tesco | Mature Cheddar Alternative | Cheddar | N | - | 150 | - | - | - |
| Coconut cont. | Vitalite | Cheese Block | Not Specified | N | - | 281 | - | 2.1 | - |
|  | Vitalite | Cheese Slices | Not Specified | N | - | 281 | - | 2.1 | - |
|  | Violife | Mature Cheddar Alternative Slices | Cheddar | N | - | - | - | 2.5 | - |
|  | Violife | Soft Cheese Alternative | Soft /cream cheese | N | - | - | - | 2.5 | - |
|  | Violife | Cheese Block | Not Specified | N | - | - | - | 2.5 | - |
|  | Violife | Cheese Grated | Not Specified | N | - | - | - | 2.5 | - |
|  | Violife | Cheese Slices | Not Specified | N | - | - | - | 2.5 | - |
|  | Violife | Prosociano Alternative | Parmesan | N | - | - | - | 2.5 | - |
|  | Violife | Mature Cheddar Alternative | Cheddar | N | - | - | - | 2.5 | - |
|  | Violife | Grated Mature Cheddar Alternative | Cheddar | N | - | - | - | 2.5 | - |
|  | Violife | Greek Style Alternative | Greek White Cheese "Feta" | N | - | - | - | 2.5 | - |
|  | Waitrose | Cheese Grated | Not Specified | N | - | - | - | - | - |
|  | Waitrose | Soft Cheese Alternative | Soft /cream cheese | N | - | - | - | - | - |

Dashed line indicates product not fortified as information not provided on the nutritional label.

Supplementary Table S1d: plant-based fish alternatives on the UK market with micronutrient fortification (per 100g). Survey completed in December 2020 omitting those only available through health-food shops.

| **Product description** | **Matrix** | **Brand** | **Fresh/frozen** | | **Organic** | | **Calcium (mg/100g)** | | **Iodine (µg/100g)** | | **Vitamin B2 (mg/100g)** | | **Vitamin B12 (µg/100g)** | | **Vitamin D (µg/100g)** | |
| --- | --- | --- | --- | --- | --- | --- | --- | --- | --- | --- | --- | --- | --- | --- | --- | --- |
| Fish Free Cakes | Soya protein | Tesco | Fresh | N | | - | | - | | - | | - | | - | |  |
| Fishless Fingers | Mushrooms | Plant Pioneers | Fresh | N | | - | | - | | - | | - | | - | |  |
| Battered Fishless Fillets | Jackfruit | Plant Pioneers | Frozen | N | | - | | - | | - | | - | | - | |  |
| Fish Goujon | Rehydrated Textured Soya | Linda McCartney | Frozen | N | | - | | - | | - | | - | | - | |  |
| Fishless Fingers | Mycoprotein | Quorn | Frozen | N | | - | | - | | - | | - | | - | |  |
| Battered Fishless Fillets | Mycoprotein | Quorn | Frozen | N | | - | | - | | - | | - | | - | |  |
| Fishless Fingers | Wheat protein | Squeaky Bean | Fresh | N | | - | | - | | - | | - | | - | |  |
| Breaded Fish Fillets | Fava bean | Morrisons | Fresh | N | | - | | - | | - | | - | | - | |  |
| Crisp-Crumbed Fishless Goujons | Jackfruit | Waitrose | Frozen | N | | - | | - | | - | | - | | - | |  |
| Breaded Fishless Lemon & Pepper Fillets | Mycoprotein | Quorn | Frozen | N | | - | | - | | - | | - | | - | |  |
| Fishless Fingers | Wheat protein | Plant Menu | Frozen | N | | - | | - | | - | | - | | - | |  |

Dashed line indicates product not fortified as information not provided on the nutritional label.

Supplementary Table S2: Nutritional composition (per 100g) of animal-based milk, yoghurt, cheese and fish from UK Food Tables ^(1)^.

| **Food Code** | **Food Name** | **Iodine** (µg/ 100g) | **Calcium** (mg/ 100g) | **Vitamin B2** (mg/ 100g) | **Vitamin B12** (µg/ 100g) | **Vitamin D** (µg/ 100g) | |
| --- | --- | --- | --- | --- | --- | --- | --- |
| **MILK** |  |  |  |  |  |  | |
| 12-512 | Milk, 1% fat, pasteurised | 30 | 123 | 0.23 | 0.9 | Tr | |
| 12-321 | Milk, Channel Islands, whole, pasteurised | 29 | 129 | 0.22 | 0.8 | 0.1 | |
| 12-322 | Milk, Channel islands, whole, summer | 24 | 129 | 0.22 | 0.8 | 0.1 | |
| 12-323 | Milk, Channel islands, whole, winter | 34 | 129 | 0.22 | 0.8 | 0.1 | |
| 12-313 | Milk, semi-skimmed, pasteurised, average | 30 | 120 | 0.24 | 0.9 | Tr | |
| 12-418 | Milk, semi-skimmed, pasteurised, summer and autumn | 20 | 118 | 0.24 | 0.8 | Tr | |
| 12-419 | Milk, semi-skimmed, pasteurised, winter and spring | 41 | 123 | 0.24 | 0.9 | Tr | |
| 12-314 | Milk, semi-skimmed, UHT | 31 | 110 | 0.18 | 0.2 | Tr | |
| 12-307 | Milk, skimmed, pasteurised, average | 30 | 125 | 0.22 | 0.8 | Tr | |
| 12-308 | Milk, skimmed, pasteurised, summer | 21 | 133 | 0.21 | 0.8 | Tr | |
| 12-309 | Milk, skimmed, pasteurised, winter | 31 | 124 | 0.22 | 0.7 | Tr | |
| 12-554 | Milk, skimmed, UHT | 25 | 102 | 0.17 | 0.6 | Tr | |
| 12-596 | Milk, whole, pasteurised, average | 31 | 120 | 0.23 | 0.9 | Tr | |
| 12-598 | Milk, whole, pasteurised, summer and autumn | 20 | 123 | 0.24 | 0.9 | Tr | |
| 12-597 | Milk, whole, pasteurised, winter and spring | 41 | 116 | 0.22 | 0.8 | Tr | |
| 12-320 | Milk, whole, UHT | 31 | 110 | 0.18 | 0.2 | 0 | |
| ***MEDIAN FOR MILK*** | | ***30*** | ***123*** | ***0.22*** | ***0.8*** | ***0.1*** |  |
| **YOGHURT** | |  |  |  |  |  |  |
| 12-534 | Fromage frais, fruit, children's, fortified | 17 | 140 | 0.29 | 0.5 | N | |
| 12-372 | Fromage frais, virtually fat free, fruit | N | 87 | 0.37 | 1.4 | Tr | |
| 12-528 | Fromage frais, virtually fat free, natural | 23 | 127 | 0.2 | 1 | Tr | |
| 12-373 | Lassi, sweetened | N | 92 | 0.21 | N | Tr | |
| 12-377 | Yoghurt, Greek style, fruit | 39 | 141 | 0.13 | 0 | 0.1 | |
| 12-555 | Yoghurt, Greek style, plain | 39 | 126 | 0.13 | 0.2 | 0.1 | |
| 12-380 | Yoghurt, low fat, fruit | 48 | 140 | 0.21 | 0.3 | Tr | |
| 12-904 | Yoghurt, low fat, hazelnut | 46 | 160 | 0.22 | 0.3 | 0.1 | |
| 12-379 | Yoghurt, low fat, plain | 34 | 162 | 0.22 | 0.3 | 0.1 | |
| 12-905 | Yoghurt, low fat, toffee | 34 | 159 | 0.33 | 0.3 | 0.1 | |
| 12-532 | Yoghurt, virtually fat free/diet, fruit | N | 140 | 0.21 | 0.3 | Tr | |
| 12-533 | Yoghurt, virtually fat free/diet, plain | 53 | 160 | 0.22 | 0.3 | Tr | |
| 12-375 | Yoghurt, whole milk, fruit | 27 | 122 | 0.16 | 0.3 | 0.1 | |
| 12-530 | Yoghurt, whole milk, infant, fruit flavour | 27 | 120 | 0.15 | 0.3 | 0.1 | |
| 12-184 | Yoghurt, whole milk, plain | 63 | 200 | 0.27 | 0.2 | 0 | |
| 12-515 | Yoghurt, whole milk, twin pot, not fruit | N | 136 | N | N | N | |
| 12-531 | Yoghurt, whole milk, twin pot, thick & creamy with fruit | N | 130 | 0.19 | 0.2 | Tr | |
| ***MEDIAN FOR YOGHURT*** | | ***37*** | ***140*** | ***0.21*** | ***0.3*** | ***0.1*** |  |
| **CHEESE** |  |  |  |  |  |  | |
| 12-540 | Cheese spread, plain | 29 | 498 | 0.36 | 0.6 | 0.2 | |
| 12-537 | Cheese spread, plain, reduced fat | 29 | 485 | 0.53 | 2 | N | |
| 12-491 | Cheese, Brie, rind only |  |  | 0.43 | 1.3 |  | |
| 12-344 | Cheese, Brie, with outer rind removed | 16 | 256 | 0.33 | 0.6 | 0.2 | |
| 12-132 | Cheese, Caerphilly | 46 | 550 | 0.47 | 1.1 | 0.2 | |
| 12-345 | Cheese, Camembert | N | 235 | 0.52 | 1.1 | 0.1 | |
| 12-548 | Cheese, Cheddar type, '30% less fat' | N | 840 | 0.53 | 1.3 | 0.1 | |
| 12-348 | Cheese, Cheddar type, half fat | N | 840 | 0.53 | 1.3 | 0.1 | |
| 12-346 | Cheese, Cheddar, English | 30 | 739 | 0.39 | 2.4 | 0.3 | |
| 12-539 | Cheese, cottage, plain | 24 | 127 | 0.24 | 0.6 | 0 | |
| 12-550 | Cheese, cottage, plain, reduced fat | 24 | 127 | 0.24 | 0.6 | 0 | |
| 12-354 | Cheese, Danish blue | 12 | 488 | 0.41 | 1.3 | 0.2 | |
| 12-152 | Cheese, Derby | 46 | 680 | 0.41 | 1.4 | 0.3 | |
| 12-494 | Cheese, Dolcelatte, rind removed | 46 | 314 | 0.46 | N | N | |
| 12-487 | Cheese, Double Gloucester | 46 | 660 | 0.45 | 1.3 | 0.3 | |
| 12-355 | Cheese, Edam | 13 | 795 | 0.35 | 2.1 | 0.2 | |
| 12-493 | Cheese, Emmental | 18 | 1025 | 0.3 | 4.1 | 0.3 | |
| 12-525 | Cheese, Feta | N | 360 | 0.21 | 1.1 | 0.5 | |
| 12-357 | Cheese, goats milk, full fat, soft, white rind | 51 | 133 | 0.39 | 0.5 | 0.5 | |
| 12-358 | Cheese, Gouda | N | 773 | 0.3 | 1.7 | 0.2 | |
| 12-164 | Cheese, Gruyere | N | 950 | 0.39 | 1.6 | 0.3 | |
| 12-496 | Cheese, Halloumi | 60 | 794 | 0.39 | 0.5 | 0.2 | |
| 12-604 | Cheese, hard, average | 41 | 707 | 0.43 | 1.6 | 0.3 | |
| 12-488 | Cheese, Lancashire | 46 | 572 | 0.43 | 1.6 | 0.2 | |
| 12-490 | Cheese, Mascarpone | 14 | 161 | 0.22 | 0.6 | 0.3 | |
| 12-360 | Cheese, Mozzarella, fresh | 18 | 362 | 0.4 | 1.7 | 0.2 | |
| 12-495 | Cheese, Paneer | N | 537 | 0.2 | N | N | |
| 12-526 | Cheese, Parmesan, fresh | 72 | 1025 | 0.32 | 3.3 | 0.3 | |
| 12-492 | Cheese, Port Salut, St Paulin type | 24 | 609 | 0.26 | 2.6 | 0.1 | |
| 12-527 | Cheese, processed, plain | 27 | 610 | 0.25 | 1.2 | N | |
| 12-549 | Cheese, processed, slices, reduced fat | 22 | 642 | 0.2 | 1 | N | |
| 12-173 | Cheese, processed, smoked | 29 | 680 | 0.27 | 0.9 | 0.2 | |
| 12-174 | Cheese, Quark | 4 | 120 | 0.3 | 0.7 | Tr | |
| 12-485 | Cheese, Red Leicester | 46 | 723 | 0.44 | 1.2 | 0.3 | |
| 12-175 | Cheese, Red Windsor | 46 | 690 | 0.37 | 1.4 | 0.3 | |
| 12-176 | Cheese, Ricotta | N | 240 | 0.19 | 0.3 | N | |
| 12-177 | Cheese, Roquefort | N | 530 | 0.65 | 0.4 | N | |
| 12-178 | Cheese, Sage Derby | 46 | 610 | 0.43 | 1.4 | 0.3 | |
| 12-551 | Cheese, spreadable, full fat, soft, white | 11 | 76 | 0.26 | 0.3 | 0.1 | |
| 12-538 | Cheese, spreadable, medium fat, soft, white | 11 | 99 | 0.34 | 0.4 | 0.1 | |
| 12-541 | Cheese, spreadable, soft white, low fat | 17 | 121 | 0.41 | 0.5 | N | |
| 12-367 | Cheese, Stilton, blue | 40 | 326 | 0.47 | 1.2 | 0.2 | |
| 12-181 | Cheese, Stilton, white | 46 | 250 | 0.37 | 1.3 | 0.2 | |
| 12-489 | Cheese, Wensleydale | 46 | 560 | 0.46 | 1.1 | 0.2 | |
| 12-486 | Cheese, White Cheshire | 41 | 516 | 0.49 | 0.9 | 0.2 | |
| 12-368 | Cheese, white, average | 41 | 544 | 0.46 | 1.6 | 0.2 | |
| ***MEDIAN FOR CHEESE*** | | ***30*** | ***544*** | ***0.39*** | ***1.2*** | ***0.2*** |  |
| **WHITE FISH** | |  |  |  |  |  |  |
| 16-369 | Cod, in batter, baked | 99 | 32 | 0.07 | 1.7 | Tr | |
| 16-426 | Cod, in batter, fried in rapeseed oil | 99 | 32 | 0.07 | 1.7 | Tr | |
| 16-428 | Cod, in batter, fried in sunflower oil, takeaway | 214 | 80 | 0.12 | 2.1 | Tr | |
| 16-368 | Cod, in batter, fried, takeaway | 214 | 79 | 0.12 | 2.1 | Tr | |
| 16-370 | Cod, in breadcrumbs, baked | 99 | 41 | 0.11 | 1 | Tr | |
| 16-444 | Fish fingers, cod, fried in rapeseed oil | 106 | 29 | 0.07 | 1.4 | Tr | |
| 16-445 | Fish fingers, cod, fried in sunflower oil | 106 | 29 | 0.07 | 1.4 | Tr | |
| 16-405 | Fish fingers, cod, grilled/baked | 117 | 32 | 0.08 | 1.5 | Tr | |
| 16-371 | Fish fingers, pollock, grilled | 47 | 23 | 0.12 | 0.3 | Tr | |
| 16-458 | Fishcakes, cod, homemade | N | 27 | 0.1 | 1.1 | 0.5 | |
| 16-407 | Fishcakes, white fish, coated in breadcrumbs, baked | 58 | 51 | 0.07 | 1.4 | Tr | |
| 16-063 | Haddock, coated in crumbs, frozen, fried in blended oil | 250 | 120 | 0.08 | 1 | Tr | |
| 16-064 | Haddock, coated in crumbs, frozen, fried in blended oil, weighed with bones and skin | 200 | 96 | 0.06 | 1 | Tr | |
| 16-062 | Haddock, coated in crumbs, frozen, raw | 210 | 120 | 0.06 | 1 | Tr | |
| 16-052 | Haddock, in batter, fried in blended oil | N | 180 | 0.13 | N | Tr | |
| 16-053 | Haddock, in batter, fried in dripping | N | 180 | 0.13 | N | Tr | |
| 16-495 | Haddock, in batter, fried in retail blend oil | N | 180 | 0.13 | N | Tr | |
| 16-055 | Haddock, in batter, fried in sunflower oil | N | 180 | 0.13 | N | Tr | |
| 16-087 | Lemon sole, goujons, baked | N | 49 | 0.08 | 1 | 0 | |
| ***MEDIAN FOR FRIED/COATED WHITE FISH*** | | ***106*** | ***51*** | ***0.08*** | ***1.40*** | ***0.25*** |  |
| **OILY FISH** | |  |  |  |  |  |  |
| 16-394 | Mackerel, flesh only, grilled | 35 | 17 | 0.37 | 9.1 | 8.5 | |
| 16-479 | Mackerel, flesh only, grilled, fillets weighed with bones and skin | 34 | 16 | 0.36 | 8.9 | 8.3 | |
| 16-393 | Mackerel, flesh only, raw | 29 | 20 | 0.3 | 8.8 | 8 | |
| 16-414 | Mackerel, flesh only, smoked | 28 | 33 | 0.37 | 10.2 | 8.2 | |
| 16-484 | Salmon, farmed, flesh only, grilled, weighed with bones and skin | 12 | 9 | 0.09 | 3 | 6.8 | |
| 16-483 | Salmon, farmed, flesh only, steamed, fillets weighed with bones and skin | 12 | 10 | 0.08 | 2.1 | 9.3 | |
| 16-420 | Salmon, pink, canned in brine, drained | 18 | 109 | 0.21 | 4.7 | 13.6 | |
| 16-418 | Salmon, red, canned in brine, drained | 22 | 164 | 0.21 | 4.9 | 10.9 | |
| 16-419 | Salmon, red, canned in brine, skinless and boneless, drained | 24 | 6 | 0.22 | 4.5 | 11.8 | |
| 16-412 | Salmon, smoked (cold-smoked) | 9 | 8 | 0.12 | 3.2 | 8.9 | |
| 16-413 | Salmon, smoked (hot-smoked) | 9 | 8 | 0.16 | 4.2 | 11 | |
| 16-485 | Salmon, wild, baked | 15 | 18 | 0.1 | 7 | 10.3 | |
| 16-486 | Salmon, wild, grilled | 14 | 18 | 0.12 | 7.6 | 10.1 | |
| 16-487 | Salmon, wild, steamed | 14 | 17 | 0.11 | 7.4 | 9.8 | |
| 50-629 | Trout, brown, steamed | 16 | 36 | 0.12 | 4.9 | N | |
| 50-630 | Trout, brown, steamed, weighed with bones | 11 | 24 | 0.08 | 3.2 | N | |
| 16-489 | Trout, rainbow, baked, flesh only, fillets weighed with bones and skin | 7 | 16 | 0.11 | 2.7 | 7.1 | |
| 16-396 | Trout, rainbow, flesh only, baked | 8 | 19 | 0.13 | 3.1 | 8.2 | |
| 16-395 | Trout, rainbow, flesh only, raw | 5 | 21 | 0.12 | 2.8 | 7.9 | |
| 16-416 | Tuna, canned in brine, drained | 12 | 10 | 0.11 | 3.4 | 1.1 | |
| 16-417 | Tuna, canned in sunflower oil, drained | 12 | 11 | 0.11 | 3.4 | 1.1 | |
| 16-400 | Tuna, flesh only, baked | 23 | 11 | 0.07 | 2.2 | 3.1 | |
| 16-399 | Tuna, flesh only, raw | 18 | 4 | 0.07 | 2.2 | 3.2 | |
| ***MEDIAN FOR OILY FISH*** | | ***14*** | ***17*** | ***0.12*** | ***4.2*** | ***8.3*** |  |

# **Supplementary Table S3**: **ingredients used to calculate the iodine content of unfortified plant-based milk, cheese, yoghurt and fish alternative products for modelling from UK food tables** ^(1)^

| **Plant-based product** | **Food Code** | **Ingredient** | **Iodine content  (µg/100 g)** |
| --- | --- | --- | --- |
| Almond-based products | 14-870 | Almonds, flaked and ground | 2 |
| Cashew-based products | 14-811 | Cashew nuts, kernel only, plain | 11 |
| Coconut-based products | 14-816 | Coconut, flesh only, fresh | 1 |
| Jackfruit-based products | 13-259 | Jackfruit, raw | 0 |
| Mushroom-based products | 13-505 | Mushrooms, white, raw | 2 |
| Oat-based products | 11-788 | Porridge oats, unfortified | 1 |
| Pea-based products | 13-130 | Peas, dried, raw | 7 |
| Mycoprotein-based products | 13-574 | Quorn, pieces, as purchased | 0 |
| Rice-based products | 11-863 | Rice, white, long grain, easy cook, raw | 2 |
| Soya-based products | 13-116 | Beans, soya, dried, boiled in unsalted water | 2 |
| Walnut-based products | 14-879 | Walnuts, kernel only | 9 |

# Supplementary Table S4: Median iodine content per child’s portion^(2; 3; 4)^ of cow’s milk products, fish, and plant-based alternative products (fortified and unfortified)

| **Category** | **Product** | **Child portion size ^(3; 5)^(g)** | **Iodine content  (µg/100 g)** | **Iodine per portion (µg/portion)** |
| --- | --- | --- | --- | --- |
| **Milk** | Cow’s Milk | 100 | 30^a^ | 30 |
|  | Fortified milk-alternative |  | 24.8^b^ | 24.8 |
|  | Unfortified milk alternative |  | 0.56^c^ | 0.56 |
| **Yoghurt** | Yoghurt from cow’s milk | 125 | 36.5^a^ | 45.6 |
|  | Fortified yoghurt alternative |  | 22.5 ^b^ | 28.1 |
|  | Unfortified yoghurt alternative |  | 0.84^c^ | 1.01 |
| **Cheese** | Cheese from cow’s milk | 20 | 29.5^a^ | 5.9 |
|  | Fortified cheese alternative |  | NA | NA |
|  | Unfortified cheese alternative |  | 0.8^c^ | 0.16 |
| **Fish** | White fish | 70 | 106 ^a^ | 74.2 |
|  | Oily fish |  | 14 | 9.8 |
|  | Fortified fish alternative |  | NA | NA |
|  | Unfortified fish alternative |  | 0.5^c^ | 0.35 |

NA: fortified product not available on the market

^a^ Median values from UK food tables for milk (skimmed, semi-skimmed, 1% and whole cow’s milk), yoghurt (low fat, Greek, Fromage Frais, whole), cheese (soft, hard, cheese spread, processed), and fish (white and oily) (see detail in Supplementary Table S2)

^b^ Median value from fortified product identified in our market survey (no fortified cheese product available on the market; Supplementary Table S1)

^c^ Calculated value based on iodine content of ingredient list (Supplementary Table S3).

# Supplementary Table S5. Dietary scenarios used to model the implications for daily iodine intake when replacing cow’s milk dairy products and fish with plant-based alternatives products.

|  | |  | **Iodine provided as percentage of iodine intake recommendation** | | |
| --- | --- | --- | --- | --- | --- |
| **Scenario** | | **Iodine provided ^a^ (µg/day)** | **Child (1-10)** (% of 90 µg/day)^(6)^ | **Child 11-14** (% of 120 µg/day)^(6)^ | **Child 15-18**  (% of 130 µg/day) |
| Reference dairy scenario | Three portions of dairy per day from cow’s milk: one portion each of milk, yoghurt, and cheese | 81.5 | 90.6% | 67.9% | 62.7% |
| I | Two portions of fortified ^f^ plant-based dairy: one portion of fortified milk alternative, one portion of fortified yoghurt alternative and one portion of unfortified cheese alternative | 53.1 | 59.0% | 44.2% | 40.8% |
| II | One portion of fortified plant-based dairy: one portion of fortified milk alternative, one portion of unfortified yoghurt and one portion of unfortified cheese alternative | 26.0 | 28.9% | 21.6% | 20.0% |
| III | Three portions of unfortified plant-based dairy alternatives: one portion each of unfortified milk, yoghurt, and cheese alternative products | 1.73 | 1.9% | 1.4% | 1.3% |
| Reference fish scenario | Two portions of fish per week: one portion of white fish and one portion of oily fish | 12.0 ^b^ | 13.3% | 10.0% | 9.2% |
| IV | Two portions of fish alternative products per week | 0.1 ^b^ | 0.1% | 0.08% | 0.08% |

^a^ calculated using data from Table S4

^b^ adjusted to daily value by dividing by 7

# References

1. Finglas P RM, Pinchen H, Berry R, Church S, Dodhia S, Powell N, Farron-Wilson M, McCardle J, Swan G. (2015) *McCance and Widdowson's The Composition of Foods integrated dataset 2015* .  : Public Health England   London (UK).

2. Infant and Toddler Forum. Portion sizes for toddlers.

3. British Nutrition Foundation. (2019) 5532 - Perfect portions for little tums: British Nutrition Foundation.

4. Department for Education. Portion sizes and food groups: Primary (4 to 10 years old).

5. First Steps Nutrition Trust. (2018) Eating well: snacks 1-4 year olds, 2nd ed.: First Steps Nutrition Trust.

6. European Food Safety Authority (2014) *Scientific Opinion on Dietary Reference Values for iodine*.
